# Changes in the proteome and secretome of rat liver sinusoidal endothelial cells during early primary culture and effects of dexamethasone

**DOI:** 10.1371/journal.pone.0273843

**Published:** 2022-09-02

**Authors:** Ruomei Li, Sabin Bhandari, Inigo Martinez-Zubiaurre, Jack-Ansgar Bruun, Ilona Urbarova, Bård Smedsrød, Jaione Simón-Santamaría, Karen Kristine Sørensen

**Affiliations:** 1 Department of Medical Biology, UiT–The Arctic University of Norway, Tromsø, Norway; 2 Department of Clinical Medicine, UiT–The Arctic University of Norway, Tromsø, Norway; 3 Department of Community Medicine, UiT–The Arctic University of Norway, Tromsø, Norway; Katholieke Universiteit Leuven, BELGIUM

## Abstract

**Introduction:**

Liver sinusoidal endothelial cells (LSECs) are specialized fenestrated scavenger endothelial cells involved in the elimination of modified plasma proteins and tissue turnover waste macromolecules from blood. LSECs also participate in liver immune responses. A challenge when studying LSEC biology is the rapid loss of the *in vivo* phenotype in culture. In this study, we have examined biological processes and pathways affected during early-stage primary culture of rat LSECs and checked for cell responses to the pro-inflammatory cytokine interleukin (IL)-1β and the anti-inflammatory drug dexamethasone.

**Methods:**

LSECs from male Sprague Dawley rats were cultured on type I collagen in 5% oxygen atmosphere in DMEM with serum-free supplements for 2 and 24 h. Quantitative proteomics using tandem mass tag technology was used to examine proteins in cells and supernatants. Validation was done with qPCR, ELISA, multiplex immunoassay, and caspase 3/7 assay. Cell ultrastructure was examined by scanning electron microscopy, and scavenger function by quantitative endocytosis assays.

**Results:**

LSECs cultured for 24 h showed a characteristic pro-inflammatory phenotype both in the presence and absence of IL-1β, with upregulation of cellular responses to cytokines and interferon-γ, cell-cell adhesion, and glycolysis, increased expression of fatty acid binding proteins (FABP4, FABP5), and downregulation of several membrane receptors (STAB1, STAB2, LYVE1, CLEC4G) and proteins in pyruvate metabolism, citric acid cycle, fatty acid elongation, amino acid metabolism, and oxidation-reduction processes. Dexamethasone inhibited apoptosis and improved LSEC viability in culture, repressed inflammatory and immune regulatory pathways and secretion of IL-1β and IL-6, and further upregulated FABP4 and FABP5 compared to time-matched controls. The LSEC porosity and endocytic activity were reduced at 24 h both with and without dexamethasone but the dexamethasone-treated cells showed a less stressed phenotype.

**Conclusion:**

Rat LSECs become activated towards a pro-inflammatory phenotype during early culture. Dexamethasone represses LSEC activation, inhibits apoptosis, and improves cell viability.

## Introduction

Endothelial cells from different vascular beds vary in their gene expression profile and structure, reflecting organ-specific functions [1, 2]. The liver sinusoidal endothelial cells (LSECs) are morphologically and functionally geared to maintain hepatic and systemic homeostasis. The cells lack an organized basal lamina and are highly perforated with transcellular, membrane-bound pores or fenestrae (approximately 50–300 nm in diameter [3]) that allow a bidirectional flow of plasma proteins, and solutes between the sinusoidal blood and hepatocytes [4, 5]. LSECs are also highly active scavenger cells [6–8], equipped with a wide repertoire of scavenger receptors and C-type lectins [9, 10] and a well-developed endocytic apparatus [11]. Studies over the last four decades have shown a pivotal role of the cells in blood clearance of many modified plasma proteins and lipoproteins, extracellular matrix molecules, viruses, and other nanoparticles [12]. In addition, LSECs participate in immune responses in the liver and are suggested to have a key role in the maintenance of liver immune tolerance [13, 14]. Paracrine signals from LSECs are essential for the normal function of other sinusoidal cells and hepatocytes, and for proper organ regeneration after partial hepatectomy [15–19]. Despite increasing awareness of the role of LSECs in liver health and disease [20–25], there are still few reports describing the LSEC proteome [9, 26–28] and information about the LSEC secretome, which is dependent on cell culture studies, remains incomplete.

One challenge with LSEC studies *in vitro* is that the cells rapidly lose their specific *in vivo* phenotype in culture, including altered gene expression of cell markers, loss of cell fenestration, cell viability, and scavenger functions [29, 30]. LSEC culture can, to some extent, be improved by co-culture with other liver cells, confirming the cells´ dependence on the sinusoidal microenvironment [31]. However, few studies have investigated the detailed molecular mechanisms behind the reported *in vitro* changes in LSEC monocellular cultures. In a study comparing the gene expression profiles of freshly isolated (0 and 2 h) rat LSECs with LSECs at 42 h post-seeding and rat lung microvascular endothelial cells it was found that rat LSECs showed significant time-dependent downregulation in growth and transcription factors essential for LSEC differentiation, as well as reduction of the LSEC markers stabilin-1, stabilin-2, FcγRIIb2, and LYVE-1, and became more like lung microvascular endothelial cells at 42 h [29].

We recently reported a study comparing the bulk transcriptomes and non-labelled proteomes of freshly isolated rat LSECs and Kupffer cells, revealing cell-specific and complementary scavenger and immune features of the two sinusoidal liver cells [9]. In the present study we have explored the rat LSEC proteome at 2 and 24 h post-seeding under pro- and anti-inflammatory conditions by quantitative proteomics, using an isobaric tandem mass tag (TMT) sixplex labeling approach combined with liquid chromatography and tandem mass spectrometry (LC-MS/MS).

The aim of the study was two-fold: 1) To reveal biological pathways and processes that are affected during early primary culture of rat LSECs at the proteome level, as this is incompletely described in the literature, and 2) examine the effect of the pro-inflammatory cytokine interleukin (IL)-1β, and the anti-inflammatory drug dexamethasone (Dex) on the cell-associated and secreted proteomes of the cells. In addition, we have looked at the effects of Dex on rat LSEC ultrastructure, scavenger function, and viability in culture. Dex is a synthetic glucocorticoid routinely used in the clinic and shows anti-inflammatory activity and modulatory effects on metabolism in many cell types [32]. Glucocorticoids, including Dex, are also frequently used as a medium supplement for *in vitro* primary cultures of different cell types. Most cells in the body express the glucocorticoid receptor (NR3C1), a nuclear receptor of the steroid /thyroid hormone receptor superfamily and regulator of glucocorticoid responses. However, the response to glucocorticoids varies between tissues, is cell type-specific in terms of the individual genes and pathways affected and may evoke disparate magnitude or direction of responses in different cell types [33–35]. Effects on endothelial cells remain insufficiently studied [36]. It is therefore of interest to know the detailed effects of these drugs in LSECs.

## Material and methods

### Animals and ethics statement

Male Sprague Dawley (Crl:CD (SD)) rats were purchased from Charles River Laboratories, (Sulzfeld, Germany) at the age of 6 weeks, and used in all experiments except in the LSEC endocytosis capacity experiments, caspase 3/7 assays, and qPCR experiments. In the latter experiments we used 6–10 weeks old male Sprague Dawley rats obtained from Janvier Labs (Le Genest-Saint-Isle, France). The rats were housed under controlled conditions (21°C ± 1°, relative humidity 55% ± 10% and 12 h light/12 h dark cycle) at the specific pathogen-free animal research facility at the University of Tromsø (UiT)–The Arctic University of Norway and were acclimatized for at least one week before the experiments. The rats were group housed (2–3 rats per cage) in 1354G Eurostandard type III conventional cages (Tecniplast, Italy) with aspen bedding (Scanbur Norway), and with nesting material, houses, and aspen bricks (Datasand Ltd, Manchester, UK) as environmental enrichment. The rats had free access to water and standard chow (RM1-E, Special Diet Service, UK). The experimental protocols were approved by the National Animal Research Authority at the Norwegian Food Safety Authority (Mattilsynet; Approval IDs: 6233, 8455, 24732) and experiments performed in compliance with the European Convention for the protection of Vertebrate Animals used for Experimental and Other Scientific Purposes. All experiments were terminal experiments, and all animals were euthanized by the following procedure: While in deep surgical anesthesia, the vena cava was cut, causing exsanguination of the animal. The anesthesia protocol is described in the next section “LSEC isolation, purification and culture”.

### LSEC isolation, purification, and culture

Rat LSECs were isolated and purified essentially as described [37]. In short, the rats were anaesthetized with either 1) a combination of ketamine hydrochloride (Ketalar 50 mg/ml; Pfizer, Norway) and medetomidine hydrochloride (Domitor vet 1mg/mL, Orion Corporation, Finland); dose of mixture: 0.15 ml Ketalar/100 g BW and 0.05ml Domitor /100 g BW, administered subcutaneously, or 2) a mixture of zolazepam/tiletamine hydrochloride 12.9/12.9 mg/ml (Zoletil forte vet, Virbac, Norway), xylazine 1.8 mg/ml (Rompun, Bayer Nordic, Norway), and fentanyl 10.3 μg/ml (Actavis, Norway); dose of mixture was 2 ml/kg body weight, administered intraperitoneally. Anesthetic depth was assessed prior to and during the operation procedure to ensure deep surgical anesthesia. After the opening of the abdomen, a catheter connected to a peristaltic pump-driven perfusion system was inserted into the portal vein, and the caudal vena cava was cut to allow outflow of buffer from the liver and exsanguination of the animal. The liver was dissected out and placed on a mesh on the top of a cylinder where run-through buffer was collected. The liver lobes were perfused free of blood with 250 ml of a calcium-free HEPES-based buffer [37], and the buffer discarded. Then the liver was perfused with 50 ml of a calcium-containing HEPES-based buffer [37] with 0.6 mg/ml collagenase type P (Worthington Biochemical Corp., Lakewood, NJ) in a recirculation system (flow rate 30 ml/min) until the cells could be released by gentle shaking. After removal of hepatocytes by low-speed differential centrifugation (50 *g*, 2 min x 3), the non-parenchymal liver cells (NPCs) in the supernatant were pelleted (300 *g*, 10 min), and loaded onto a two-step 25%-45% Percoll gradient (GE Healthcare, Uppsala, Sweden), and centrifuged at 1350 *g* for 30 min. Cells at the Percoll 25%-45% interface were collected and LSECs purified by selective adherence: first, the cell suspension was seeded onto non-coated tissue culture plates and incubated at 37°C for 30 min, leaving Kupffer cells but not LSECs to attach to the substrate; then the non-adherent cells, highly enriched in LSECs were collected and used in the experiments. The average yield of LSECs for proteomics experiments was approximately 60x10^6^ cells per liver (body weight: 250–300 g), and LSECs from two rat livers were pooled in each proteomics experiment. For isolation of LSECs for some experiments, including the qPCR experiments, caspase 3/7, and endocytosis capacity assays, we used Liberase^TM^ (Roche, Cat. No 05401127001), 0.03 mg/ml in calcium-containing perfusion buffer instead of Collagenase type P, with similar cell yields and purity.

Purified LSECs were seeded on bovine type I collagen (2.9 μg/ml; Advanced BioMatrix, San Diego, CA) coated tissue culture plates (100 mm; Sarstedt, Nümbrecht, Germany), or 24-well tissue culture plates (Sarstedt) at a density of 0.3×10^6^ cells/cm^2^ in Dulbecco´s modified Eagle´s cell culture medium (DMEM, Sigma, St. Louis, MO). The cells were first incubated for 30 min (37°C, 5% CO_2_, 5% O_2_), then washed with prewarmed medium to remove debris and non-attached cells, before further incubation in DMEM supplemented with Insulin-transferrin-sodium selenite supplement (ITS, Sigma, Cat. No I3146), ascorbic acid (62 μg/ml, Sigma, Cat. No A4403), sodium pyruvate (1mM, Gibco, Cat. No 11360039), glutamax (2 mM of L-alanyl-L-glutamine dipeptide, Gibco, Cat. No 35050–038), penicillin (100 units/ml) and streptomycin (0.1 mg/ml) (Sigma), with or without the addition of 1 μg/ml of Dex (Fortecortin^TM^ Inject 4 mg/ml, Merck Serono GmbH, Darmstadt, Germany) (dose as in [38]), and/or 100 U/ml of human recombinant IL-1β (PeproTech, NJ, Cat. No 200-01B) [39]. All cultures were incubated at 37°C, in 5% CO_2_ and low oxygen (5% O_2_), as recommended for LSECs in [30]. DMEM with supplements was used in all experiment unless otherwise stated.

The purity of LSEC cultures was assessed by scanning electron microscopy (SEM), and immune labeling for the LSEC marker stabilin-2 [9, 29]. Cells purified for quantitative proteomics (n = 3; each biological replicate representing the pooled LSECs from two rat livers) showed 93.2% (SD = 1.5%) fenestrated endothelial cells, which is the hallmark of LSECs [40]. Cells for SEM were from the same cell isolation as used for proteomics and were seeded at similar density as for the proteomics experiments and incubated and washed in the same way and fixed after 2h (the SEM preparation protocol is included in the Material and Methods section “Scanning electron microscopy”). Parallel LSEC cultures were also immune labelled with an antibody to whole rat stabilin-2 [41] resulting in 91.8% (SD = 0.7%) positive cells (n = 3). Contaminating cells were identified as Kupffer cells (CD163 positive) and hepatic stellate cells (glial fibrillary acidic protein, GFAP, positive) by immune labeling with mouse monoclonal anti-rat CD163 (Bio-Rad, Kidlington, UK, Cat. No MCA342GA), and rabbit polyclonal anti-rat glial fibrillary acidic protein (GFAP) (Dako, Glostrup, Denmark, Cat. No Z0334).

The purity of LSEC cultures for Luminex multiplex immunobead analysis of cytokine secretion to supernatants was also assessed by SEM. The percentage of LSECs (*i*.*e*., fenestrated endothelial cells) in cultures incubated for 24 h were 94.0% (SD = 3.5%) in the absence of Dex (n = 3), and 97.3% (SD = 1.2%) in the presence of Dex (n = 3).

### Proteomics sample preparation and identification of proteins

**Fig 1:**
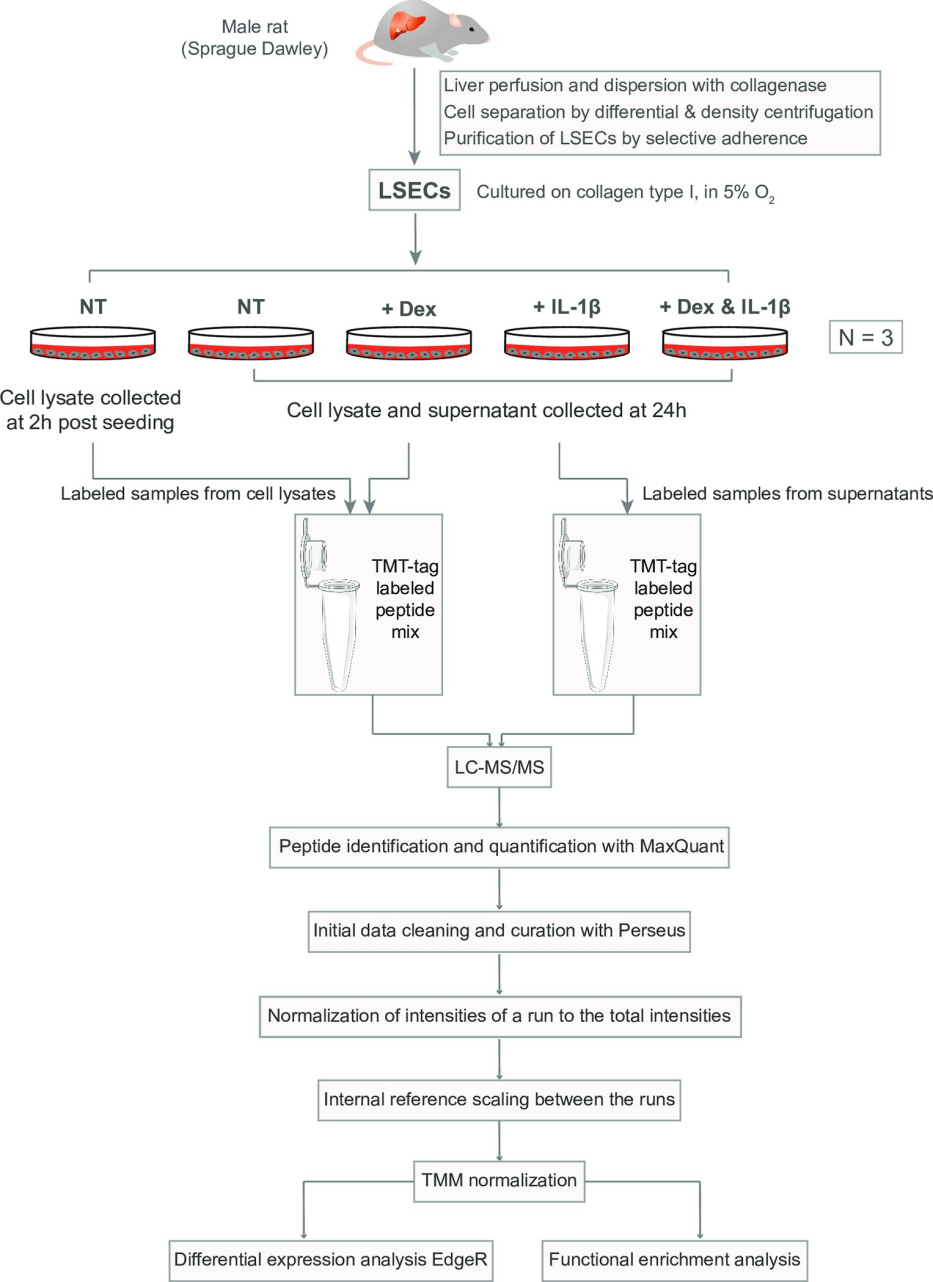
Overview of the experimental setup for the TMT proteomics study. The figure shows the experimental setup for the quantitative proteomics experiment. The whole experiment was repeated three times with cells from different rats. Each biological replicate included pooled LSECs from 2 rat livers. The dose of Dex was 1 μg/ml [38]. The dose of IL-1β was 100 U/ml, which was chosen after testing the effect of different doses on IL-6 production in LSECs in an ELISA assay (S1 Fig). NT: not treated with Dex, or IL-1β.

An overview of the experimental setup for the quantitative TMT proteomics experiments is shown in Fig 1.

Serum-free supernatants of LSECs cultured for 24 h were collected and passed through a filter (pore size 0.2 μm) to remove debris, then concentrated on Vivaspin columns (3kD cut-off, Sartorius AG). Concentrated samples were washed 3 times with PBS on the column and the volume was reduced to 0.2 ml. Cellular protein extracts of LSEC cultures incubated for either 2 or 24 h were prepared according to the protocol provided in the TMTsixplex^TM^ Isobaric Mass Tagging Kit (Thermo Fisher Scientific, Waltham, MA) with the following modification: Denaturing reagent was 5% sodium deoxycholate in 100 mM TEAB (triethylammonium bicarbonate). Protein concentrations of concentrated supernatant and cellular extracts were measured with Direct Detect^TM^ Infrared Spectrometer (Millipore).

Proteins in cell extracts and supernatants were reduced according to the protocol provided in the TMTsixplex^TM^ Isobaric Mass Tagging Kit, except that the reducing reagent was 5 mM dithiothreitol (Sigma) instead of tri(2-carboxyethyl)-phosphine. Proteins were precipitated with acetone and the pellet was collected by centrifugation at 16000 *g* for 10 min. The protein pellet (25 μg) was resuspended in 2 M urea, 50 mM TEAB. Proteins were digested for 6 h by 1:100 (w/w) lysyl endopeptidase (Fujifilm Wako Chemicals Europe GmBH, Neuss, Germany). The samples were diluted to 1 M urea and digested overnight with 1:20 (w/w) trypsin (V511A, Promega Corporation, WI). Peptides from each sample were labelled with the TMTsixplex^TM^ Isobaric Mass Tagging Kit according to the manufacturer’s protocol. OMIX C18 tips (Varian, Inc., Palo Alto, CA) were used for sample cleanup and concentration. Peptide mixtures containing 0.1% formic acid were loaded onto Thermo Fisher Scientific EASY-nLC1000 system and EASY-Spray column (C18, 2 μm, 100 Å, 50 μm, 50 cm). Peptides were fractionated using a 2–100% acetonitrile gradient in 0.1% formic acid over 180 min at a flow rate of 250 nl/min. The separated peptides were analyzed using a Thermo Scientific Q-Exactive mass spectrometer. Data was collected in data-dependent mode using a Top10 method. Raw data were processed using MaxQuant (version 1.5.0.30), and proteins identified using the integrated Andromeda search engine. Tandem mass spectrometry (MS/MS) data were searched against the UniProt Rattus norvegicus (Rat) reference proteome [42]. A false discovery rate (FDR) ratio of 0.01 was needed to give a protein identification.

### Data analysis

The resulting output from MaxQuant was pre-processed with Perseus (version 1.6.10.43). The generated list of proteins was filtered to remove protein hits that were annotated as only identified by site, contaminants, and reverse hits in Perseus. The annotation of the protein IDs to their corresponding gene symbols was manually curated with the UniProt Knowledgebase (UniProtKB; [42]). Intensity values of redundant gene symbols were summed before doing the differential expression analysis. The tag-reporter intensity corrected output from the MaxQuant was used for protein quantification. Each single TMT run contained all the experimental groups from one rat (= one biological replicate; see the experimental setup in Fig 1), and the reporter intensity was therefore dependent both on the run and the rat. The runs were separately scaled, followed by internal reference scaling normalization [43, 44]. Finally, the data was normalized for compositional bias using edgeR TMM normalization (Bioconductor [45]). These normalized intensities were then used in differential protein expression analysis using the edgeR-limma workflow [46].

In addition, the differential protein expression in the supernatant datasets was checked by adjusting the normalized data for the total amount of histone and ribosomal proteins. The result displayed no significant difference prior to and after scaling for histone and ribosomal proteins in terms of the differential expression analysis (not shown). Therefore, supernatant datasets without additional histone and ribosomal protein scaling were used for the subsequent analysis and illustration as we wanted to keep the normalization steps to a minimum to prevent inadvertent bias due to inherent variability in the expression of histone and ribosomes in our data.

### Gene set enrichment analysis (GSEA)

Protein IDs were converted to gene symbol before loading into GSEA-4.1.0 [47, 48]. The normalized reporter intensity data were fed for the functional enrichment analysis. Signal-to-noise weighted scoring schemes were used for the enrichment statistics. Gene set permutation of 10,000 times was done to obtain the null distribution of p-values. Gene sets with an FDR ≤ 0.05 were considered significantly enriched.

### Endocytosis experiments

LSECs (0.3×10^6^ cells/cm^2^ in collagen-coated 24-well plates) were cultured as described under “LSEC isolation, purification and culture”, with or without supplementation of 1 μg/ml Dex. Formaldehyde-denatured bovine serum albumin (FSA), prepared as described in [49] was labeled with either fluorescein isothiocyanate (FITC) by incubating FSA and FITC in sodium carbonate buffer (0.5 ml/l, pH 9.5) at a protein-dye ratio of 4:1 at 4°C overnight, and dialyzed against PBS, or with carrier-free Na^125^I using Iodogen as an oxidizing agent, as described by the manufacturer (Pierce Chemicals, Rockford, IL). FSA labeled with ^125^I was separated from unbound ^125^I on a PD-10 column (GE Health, Uppsala, Sweden). The resulting specific radioactivity was approximately 10^6^ counts per minute per μg protein.

Three sets of endocytosis experiments were carried out. In the first set of experiments, we measured the uptake and intracellular degradation of trace amounts of radiolabeled FSA during a 2 h period as described in [50]. LSEC cultures were incubated with ^125^I-FSA (0.1 μg/ml) in Roswell Park Memorial Institute (RPMI-1640) medium (Sigma) with 1% human serum albumin (Octapharma, Heidelberg, Germany) for 2 h at 37°C in 5% O_2_ and 5% CO_2_. The supernatant was then removed and intact proteins in the supernatant pelleted with 20% trichloroacetic acid (Merck), while cell cultures were washed with cold PBS and lysed in 1% sodium dodecyl sulphate before measuring cell-associated, and acid-soluble radioactivity in supernatants (the latter representing degraded ligand [50]) in a gamma counter. Each experiment was done in triplicate and repeated with 3 biological replicates. For measurements of cell numbers in cultures, 5 areas per culture, and per treatment were imaged before starting the endocytosis experiments, using a Nikon inverted microscope (Nikon, Tokyo, Japan) equipped with a Zeiss AxioCam MRc digital camera (Carl Zeiss, Göttingen, Germany). Cell numbers were counted manually with Fiji software [51].

In the second set of endocytosis experiments, we measured the LSEC capacity of FSA uptake, using the same protocol as described above, with the exception that 0.1 μg/ml ^125^I-FSA was mixed with increasing concentrations (0–20 μg/ml) of non-radiolabeled FSA, then incubated for 2 h at 37°C in 5% O_2_, 5% CO_2_ (each experiment done in triplicate, and repeated with 3 biological replicates). In each experiment, parallel cultures with similar numbers of cells as in the endocytosis experiments were fixed at the same time point. The cell nuclei were stained with DAPI (Sigma-Aldrich Cat. No D8417) and imaged with Celldiscoverer7 (Carl Zeiss), then counted with Fiji software. The uptake of FSA in pg per cell was then calculated from the total ligand uptake per culture divided by cell number.

In the third set of endocytosis experiments, LSEC cultures were incubated with FITC-labelled FSA and examined by confocal microscopy to study ligand uptake in individual LSECs. The cultures were incubated with 20 μg/ml of FITC-FSA in RPMI-1640 with 1% human serum albumin for 30 min, then the medium with ligand was removed and the cells incubated further in medium alone for another 1.5 h before fixation of cells in 4% formaldehyde in PBS, pH 7.2. Cell nuclei were stained with DAPI (Sigma-Aldrich), and the cultures imaged in a Zeiss LSM800 confocal laser scanning microscope (Carl Zeiss) equipped with a 40x water objective.

### Scanning electron microscopy (SEM)

LSECs (0.3×10^6^ cells/cm^2^) were seeded in collagen-coated 24-well plates and treated as described under “LSEC isolation, purification, and culture” before fixation in McDowell´s fixative for electron microscopy [52] at 2 or 24 h after seeding. Fixed cultures on tissue culture plastic were stamped out from the plate and processed for SEM using the protocol in [9]. In short, the cells were washed in PHEM buffer, post-fixed in 1% tannic acid, then in 1% osmium tetroxide in double-distilled water, dehydrated in a graded ethanol series (30–100%), chemically dried in hexamethyldisilazane (Sigma Aldrich; cells for morphology analysis and sieve plate counts), or critical point dried (cells for purity tests for proteomics), mounted on aluminum stubs, and sputter coated with gold/palladium alloy. Specimens were imaged in a Zeiss Sigma field emission scanning electron microscope (Carl Zeiss, Oberkochen, Germany), run at 2 kV. High-resolution overview images were taken from 3–5 different regions per culture, and high magnification images obtained within these areas.

When comparing the number of sieve plates per cell area in rat LSEC cultures incubated for 2 or 24 h, fenestrae were defined as open trans-cytoplasmic holes < 500 nm in diameter, whereas holes above this size were defined as gaps. Sieve plates were defined as clusters of ≥ 5 fenestrations. The image analysis was done in a semi-quantitative way, where the area covered by cells in each image was divided with a grid into squares of 20 μm x 20 μm (400 μm^2^) using Image J, and the number of sieve plates was counted per square. More than 100 squares of 400 μm^2^ were analyzed from each culture. When establishing the method for this analysis, 35 randomly selected squares were evaluated independently by three experienced LSEC researchers (RL, KKS, BS). Without counting the actual number of sieve plates, each researcher was asked to score the number of sieve plates within each square as high, medium, and low according to their experience. More than 90% consensus was reached between the researchers. The number of sieve plates in the areas scored as high, medium, or low fenestrated were then counted, and the criteria of each category was set as follows: 1) Low: 0–4 sieve plates/400 μm^2^, 2) medium: 5–14 sieve plates/400 μm^2^, and 3) high: ≥ 15 sieve plates/400 μm^2^.

### Cell viability assay

LSECs (0.3×10^6^ cells/cm^2^) were seeded in collagen-coated 24-well plates and treated as described under “LSEC isolation, purification and culture”. At 2 or 24 h, the fluorescent dye mixture from the Live/Dead Cell Imaging Kit (Thermo Scientific) was added to each culture and the cells incubated according to the manufacturer’s instructions. The cultures were imaged in a Nikon inverted microscope, and the number of positively stained cells counted with Fiji software and corrected manually when two cells were closely attached to each other.

### Caspase 3/7 assay

LSECs, 0.1×10^6^ per well, were seeded in 96 well plates (Corning Costar® Cat. No 3610). Cells were washed after 40 min, then incubated with 0.1, 1, or 10 μg/ml of Dex, or without Dex for 24 h. The positive control was established by replacing the medium in non-treated cultures at 20 h with medium containing 100 ng/ml of recombinant human Fas Ligand protein (rhsFasL, Abcam, Cat. No ab157085), then incubation was continued for 4h. At 24h, Caspase-Glo® 3/7 Assay reagents (Promega Corporation, Cat. No G8090) were added to all wells and results read by CLARIOstar Plus microplate reader (BMG Labtech GMbH, Ortenberg, Germany) after 1 h. The experiment was done in triplicate and repeated in 4 biological replicates.

### Enzyme-linked immunosorbent assay (ELISA)

IL-6 production was measured after treatment of cells with Dex, or human recombinant IL-1β (PeproTech, Cat. No 200-01B). Supernatants were harvested from LSECs (0.3×10^6^ cells/cm^2^ seeded per well in 24-well plates) at specified time points, and the concentration of IL-6 was determined with the Rat IL-6 DuoSet ELISA kit (R&D Systems, Cat. No DY506). When titrating the IL-1β dose for the proteomics experiments the IL-6 production was measured at 6, 18, and 24 h (S1 Fig); 100 U/ml gave the highest IL-6 production at 18 and 24 h relative to 6 h and were used in the proteomics experiments.

### Luminex–multiplex immunobead assay

LSECs were plated in 24 well plates (0.3×10^6^ cells seeded per cm^2^), washed after 30 min, and cultures incubated further in equal volumes of medium, in the presence or absence of 1 μg/ml Dex for 24 h. Then the supernatants were collected, filtered, and frozen at -70°C until analysis, and the cells fixed for SEM. The concentration of IL-1β, IL-10, IL-6, monocyte chemoattractant protein-1 (MCP-1; CCL2), MCP-3 (CCL7), and macrophage inflammatory protein-1alfa (MIP-1α) in supernatants was measured with the Rat Custom ProcartaPlex 6-plex Kit (Thermo Fisher) following the ProcartaPlex Multiplex Immunoassay user guide MAN0017083. Specific luminescence in samples and standards were differentiated and analyzed by Luminex 100/200 (Thermo Fisher). MIP-1α values were out of range (too high) and excluded from the analysis.

### RNA isolation and cDNA synthesis

LSEC cultures were treated with 1 μg/ml Dex for 6 and 22 h, and time-matched non-treated cells in parallel cultures used as control. The cells were collected with RNAprotect Cell Reagent (Qiagen, Cat. No 76526) following the manufacturer´s protocol and the total RNA extracted with the RNeasy Mini Kit (Qiagen, Cat. No 74624), using QIAshredder (Qiagen, Cat. No 79656) to homogenize the cells and the gDNA eliminator columns, then eluted in 30 μl RNase-free water and stored at −70°C until cDNA synthesis was performed. RNA purity and concentration was assessed with NanoDrop (A260/A280 was ≅ 2 in all samples). Integrity of total RNA was determined by Bioanalyzer 2100 from Agilent, and RNA integrity numbers above 8 were accepted. cDNA libraries were prepared with the QuantiTect Reverse Transcription Kit (Qiagen, Cat. No 205311) using 1 μg per reaction, 3 reactions per samples were run, and cDNA pooled, and diluted 4x with RNase free water.

### Quantitative reverse transcription PCR (qPCR) and data analysis

All primers were designed using the PrimerQuest tool by Integrated DNA Technologies (S1 Table), choosing exon-exon locations for forward and reverse primers whenever possible. The efficiency of the primers was tested on gBlocks (Integrated DNA Technologies, S2 Table). PCR reactions were performed using FastStart Essential DNA Green Master (Roche, Cat. No 06402712001) with 5μl cDNA. A reference gene panel for rat genes (TATAA Biocenter AB, Cat. No A103) was used to find suitable reference genes. The qPCR data was analyzed using Genex7 software (MultiD Analyses AB), reference genes selected by Normfinder [53], and the combination of actin beta (*Actb*), beta-2 microglobulin (*B2m*), and hypoxanthine guanine phosphoribosyl transferase (*Hprt*) used for normalization. Data was normalized first by correcting the efficiency of each assay, then normalized to the reference genes in Normfinder, followed by calculation of the relative mRNA quantities for the individual genes at 6 and 22 h in Dex-treated and non-treated samples using the value for the 6 h non-treated sample as reference.

### Statistical analysis, and visualization

The initial data annotation and filtrations were done in the Perseus environment (version 1.6.10.43). TMT data processing and normalization (as described under “Data analysis”), and the subsequent differential expression analysis were performed in the R/Bioconductor environment [54]. The normalized intensities were subjected to the exact test implemented in the edgeR package and proteins that showed |log_2_ FC| ≥ 0.5 and FDR ≤ 0.05 were deemed significantly different. The gene enrichment analysis was performed in the GSEA-4.1.0 software [47, 48], and the gene sets with FDR ≤ 0.05 were identified as significantly enriched.

Statistical analysis of qPCR data was performed using GraphPad Prism version 9.3.1 for macOS (GraphPad Software, San Diego, CA). Gaussian distributed data were analysed using repeated-measures one-way ANOVA with Greenhouse-Geisser correction, and non-Gaussian distributed data analysed using Friedman test; p-value ≤ 0.05 was considered significant. Other statistical analyses were performed using SPSS version 28.0.0.0 (IMB SPSS Statistics).

Figures were generated using the R packages factoextra, ggplot2, ggpubr, pheatmap, and the plugin EnrichmentMap from Cytoscape, InstantClue, and Microsoft Office Excel. Panels were made in Adobe Illustrator and GraphPad Prism.

### Proteomics data availability

The mass spectrometry proteomics data have been deposited in the ProteomeXchange Consortium via the PRIDE [55] partner repository with the dataset identifier PXD029241. The whole processed proteome is included in S1 Dataset.

## Results

### Global information about the rat LSEC proteome in early primary culture

**Fig 2:**
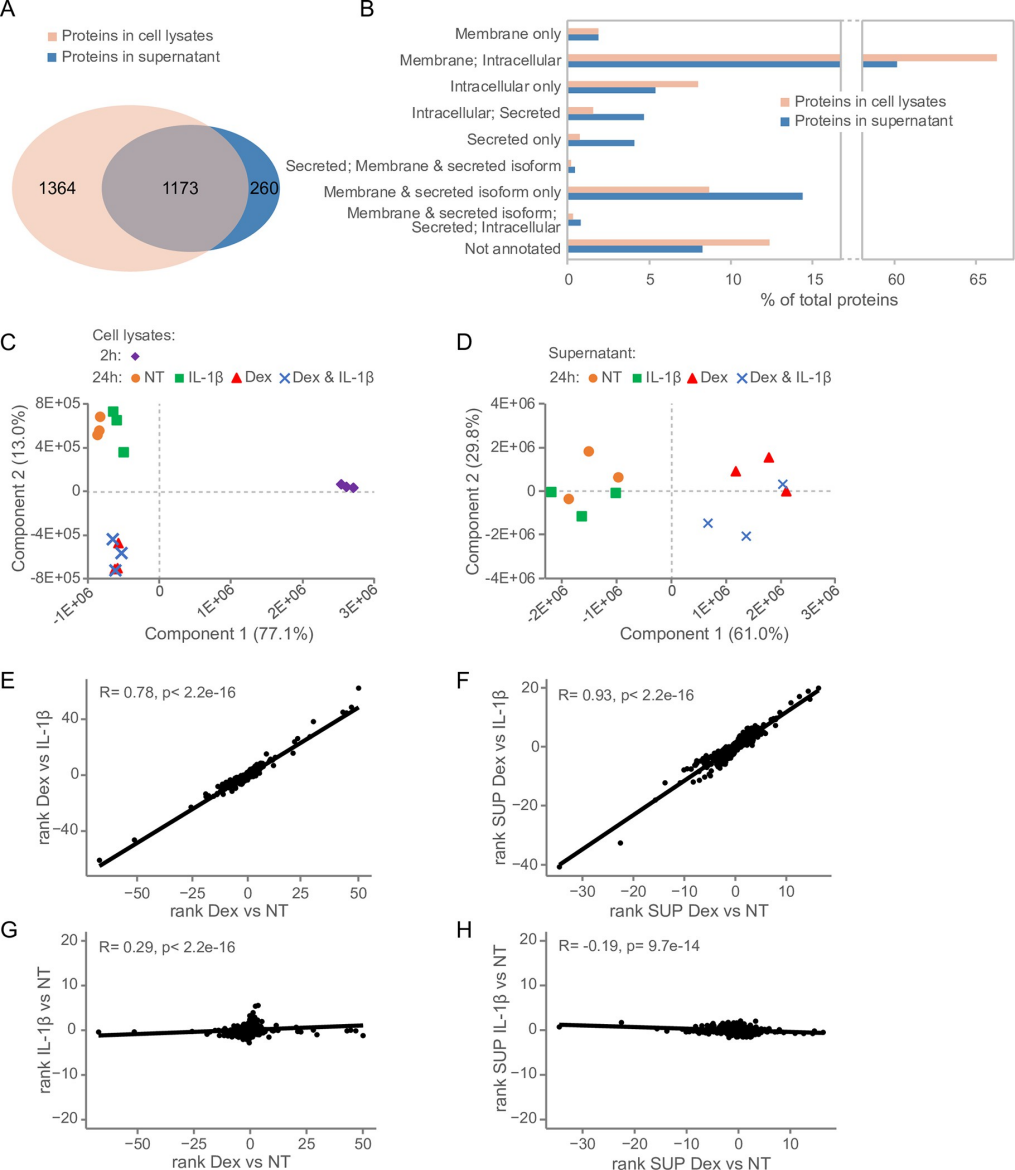
Global characterization of proteins in cell lysates and supernatants from rat LSEC primary cultures. (A) Venn diagram showing the number of proteins identified in cell lysates and supernatants of LSEC cultures and overlapping protein expression in the two sample types. (B) Protein composition of cell lysates and supernatants of LSECs cultured for 24 h. Results are presented as percent of total protein in the respective datasets and annotated to the major protein categories in Human Protein Atlas [56]. (C) Non-scaled principal component analysis (PCA) plot of proteomes of cell lysates of LSECs cultured for 2 or 24 h with Dex, IL-1β, a combination of Dex and IL-1β, or with no treatment (NT). (D) Non-scaled PCA plot of proteomes obtained from supernatants of LSECs cultured for 24 h with Dex, IL-1β, or a combination of Dex and IL-1β, or with NT. (E-H) Scatter plots showing ranked proteomes in response to Dex and IL-1β compared to NT control cultures at 24 h. The plots in E (proteomes from cell lysates) and F (proteomes from supernatants) show the correlation between the Dex vs IL-1β comparison and the Dex vs 24 h NT comparison. The plots in G (proteomes from cell lysates) and H (proteomes from supernatants) show the correlation between the Dex vs 24 h NT comparison and the IL-1β vs 24 h NT comparison.

The overview of the experimental setup is presented in Fig 1 (Material and Methods). We quantitatively catalogued 2537 protein IDs in the cell lysates (*i*.*e*., cell-associated proteins) and 1433 proteins in the filtered supernatants of the LSEC cultures in the 3 experiments (S1 Dataset). LSEC supernatants were harvested at 24 h only, whereas cell lysates were collected from both 2 h and 24 h cultures. Approximately 18% (260) of the proteins identified in the supernatants were not identified in the cell lysates (Fig 2A), and the supernatants contained more of proteins/protein isoforms predicted to be secreted (Fig 2B; detailed in S1 Dataset) using the protein class annotation in the Human Protein Atlas [56].

Principal component analysis (PCA) clustered the cell-associated proteomes into three groups (Fig 2C): 1) Samples from freshly isolated LSECs cultured for 2 h with no treatment, 2) samples from LSECs cultured for 24 h with no treatment or with IL-1β alone, and 3) samples from LSECs cultured for 24 h with Dex alone or with Dex plus IL-1β. The proteomes from the culture supernatants (harvested after 24 h and including all identified proteins in the supernatants) clustered into two groups in the PCA plot (Fig 2D): 1) Samples from LSECs cultured with no treatment or with IL-1β alone, and 2) samples from LSECs incubated in the presence of Dex alone, or with Dex plus IL-1β.

Dex induced significant changes both in the LSEC cell-associated proteome and the proteome from the LSEC supernatants, while the effect of IL-1β was low as illustrated in the scatter plots in Fig 2E–2H. We here compared the ranks obtained from the pairwise comparison of protein expression in cultures with different combinations of treatments. The high correlation across the protein ranks obtained from the pairwise comparison between the proteomes of cells (Fig 2E, R = 0.78), or supernatants (Fig 2F, R = 0.93) of [Dex-treated LSECs vs 24 h non-treated LSECs] and [Dex-treated LSECs vs IL-1β-treated LSECs] suggests that the expression level of proteins in IL-1β stimulated cultures were very similar to non-treated cultures. Furthermore, the low correlation across the protein ranks obtained from the pairwise comparison between the proteomes of cells (Fig 2G, R = 0.29), or supernatants (Fig 2H, R = -0.19) of [Dex-treated LSECs vs 24 h non-treated LSECs] and [IL-1β treated LSECs vs 24 h non-treated LSECs] indicates that Dex induced a significantly different proteomics response in LSECs than IL-1β.

Since LSECs exposed to IL1-β showed highly similar protein expression levels to non-treated LSECs at 24 h (S1 Dataset) we have focused our further analysis on the effect of Dex on the LSEC proteome.

### Rat LSECs developed a pro-inflammatory phenotype and showed major changes in metabolic pathways during the first 24 h in culture

To unravel proteome changes that occur in the early primary culture of rat LSECs, we first compared the protein expression profiles of the cell lysates from LSECs cultured for 24 h in the presence (n = 3), or absence of Dex (n = 3) with the profile of non-treated LSECs cultured for 2 h (n = 3).

**Fig 3:**
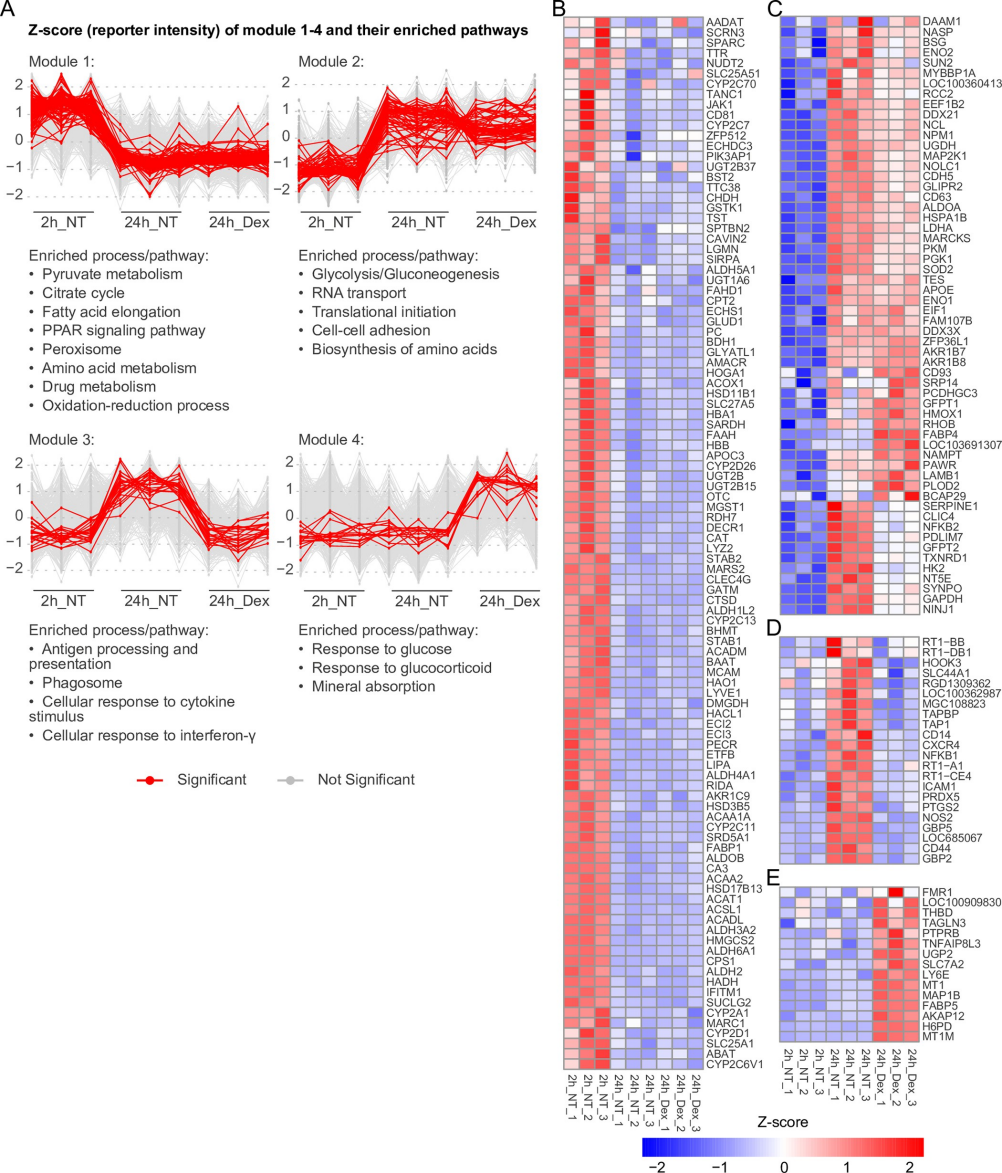
Cluster analysis of the cell-associated proteomes of 2 h vs 24 h LSEC cultures. The figure shows proteins that are significantly differentially expressed between rat LSEC cultures at 2 and 24 h post seeding. (A) Row-wise k-means clustering on the whole cell lysate proteome of non-treated LSECs (2h_NT, 24h_NT) and dexamethasone-treated LSECs (24h_Dex) resulted in four different modules. Proteins within each module 1–4 showed a similar expression pattern. These are illustrated in the parallel line (profile) plots with corresponding Z-scores (log_**2**_ normalized TMT reporter intensities). The list of proteins from each module with at least |log_**2**_ FC| ≥ 0.25, and FDR ≤ 0.05 in at least one pairwise comparison between the 2h_NT, 24h_NT, or 24h_Dex group were fed into DAVID (v 6.8) [57, 58] for functional annotation and enrichment analysis. The enriched processes/pathways associated with each module are listed below the corresponding parallel line plot. In each profile plot significantly up- and downregulated proteins belonging to each module are highlighted in red (FDR ≤ 0.05, and |log_**2**_ FC| ≥ 0.5; differential expression analysis using edgeR exact T-test), and the other proteins are shown in grey. The significantly regulated proteins are named in the heatmaps in B-E. (B) Row-scaled heatmap of proteins downregulated at 24 h in the presence and absence of Dex. (C) Row-scaled heatmap of proteins upregulated at 24 h in the presence and absence of Dex. (D) Row-scaled heatmap of proteins upregulated at 24 h in non-treated cultures only. (E) Row-scaled heatmap of proteins upregulated at 24 h in Dex-treated cultures only.

Row-wise k-means cluster analysis on the cell-associated proteomes of non-treated (2 h, 24 h) and 24 h Dex-treated LSECs resulted in four modules, where proteins within each module showed a similar pattern of expression (Fig 3A). Fig 3A also shows enriched processes and pathways that are associated with each module (resulting from functional enrichment analysis in DAVID). The significantly up-, or downregulated proteins within each of the four modules, resulting from differential expression analysis using edgeR are shown in the row-scaled heatmaps (Z-score values) in Fig 3B–3E.

Maintaining rat LSECs in culture for 24 h evoked substantial changes in the cell proteome. The majority of these changes were not significantly altered by Dex treatment (Fig 3A—modules 1 and 2). Module 1 contained the proteins that were downregulated at 24 h compared to 2h both in the presence and absence of Dex (Fig 3A and 3B). Functional enrichment analysis of proteins in this module showed downregulation of pyruvate metabolism, citrate cycle, oxidation-reduction processes, fatty acid elongation, amino acid metabolism, peroxisome, and PPAR signaling pathway.

Module 2 (Fig 3A and 3C) contained proteins that were upregulated at 24 h in all cultures. Enriched processes and pathways were glycolysis/gluconeogenesis, mRNA transport and initiation of translation, biosynthesis of amino acids, and cell-cell adhesion.

Module 3 (Fig 3A and 3D) contained proteins that were upregulated at 24 h in LSEC cultures without Dex but repressed in the presence of Dex. Significantly altered proteins in this cluster were closely associated with immune functions and inflammatory processes.

Module 4 (Fig 3A and 3E) included proteins that were upregulated at 24 h only in presence of Dex. Enriched processes and pathways were linked to responses to glucose and glucocorticoids, and mineral absorption.

### Dex suppressed the induction of an inflammatory-like phenotype in primary LSEC cultures

**Fig 4:**
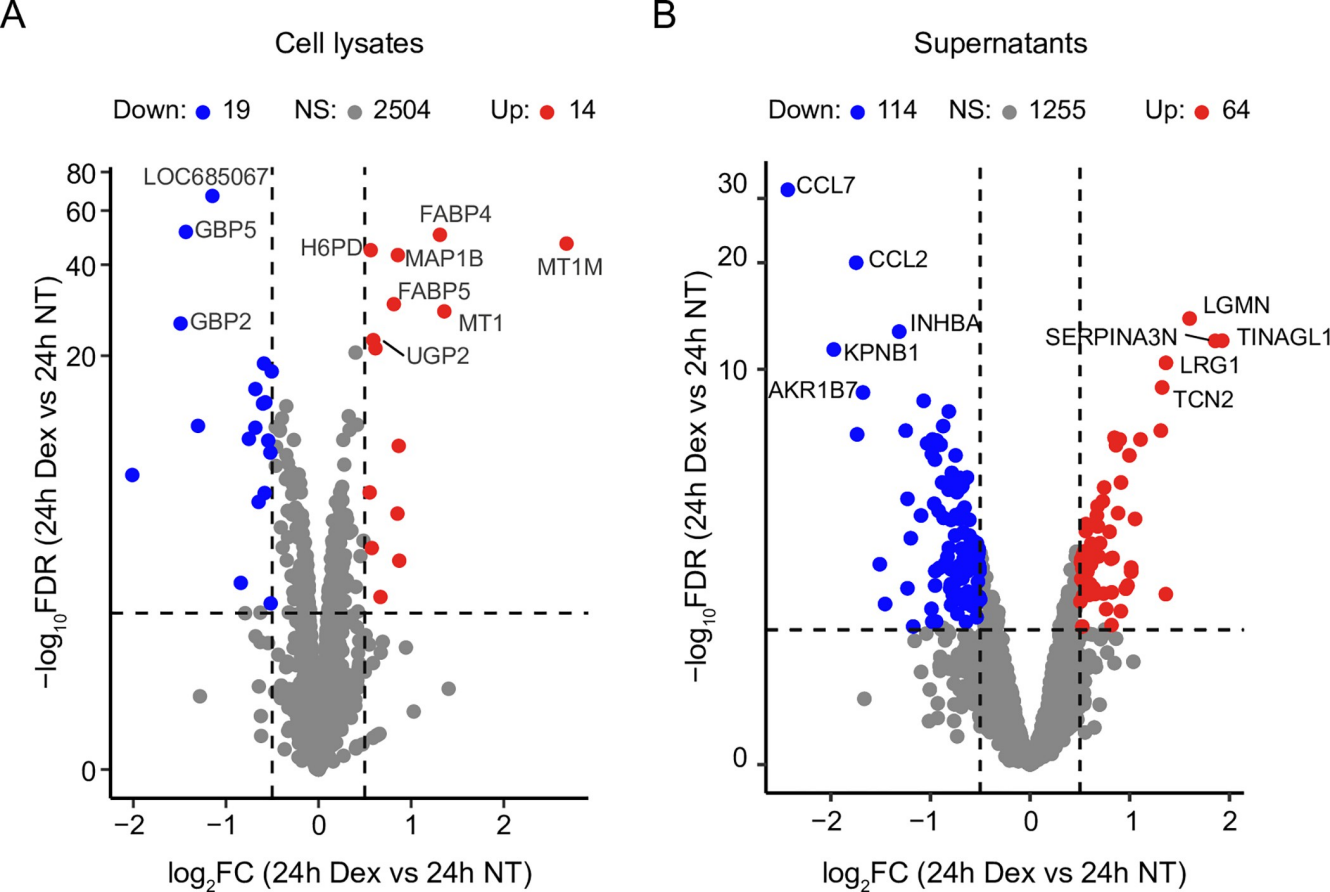
Comparison of the proteomes of cell lysates and supernatants of 24 h LSEC cultures incubated with or without Dex. Volcano plots illustrating differently expressed proteins in (A) the LSEC cell-associated proteome at 24 h ± Dex, and (B) the LSEC supernatants ± Dex. The Top 10 differentiated proteins are illustrated with symbols. Significance level: FDR ≤ 0.05, and |log_**2**_ FC| ≥ 0.5. NS, not significant.

To evaluate the effect of Dex on LSECs in more detail, we compared the proteomes from Dex-treated and non-treated cultures at 24 h focusing both on the cell-associated proteome and proteins in culture supernatants. Approximately 1.2% (33) of the cell-associated proteins were differentially expressed in the presence of Dex (Fig 4A), whereas 19% (178) of all proteins in the supernatants were significantly different between Dex-treated and non-treated cultures (Fig 4B).

**Fig 5:**
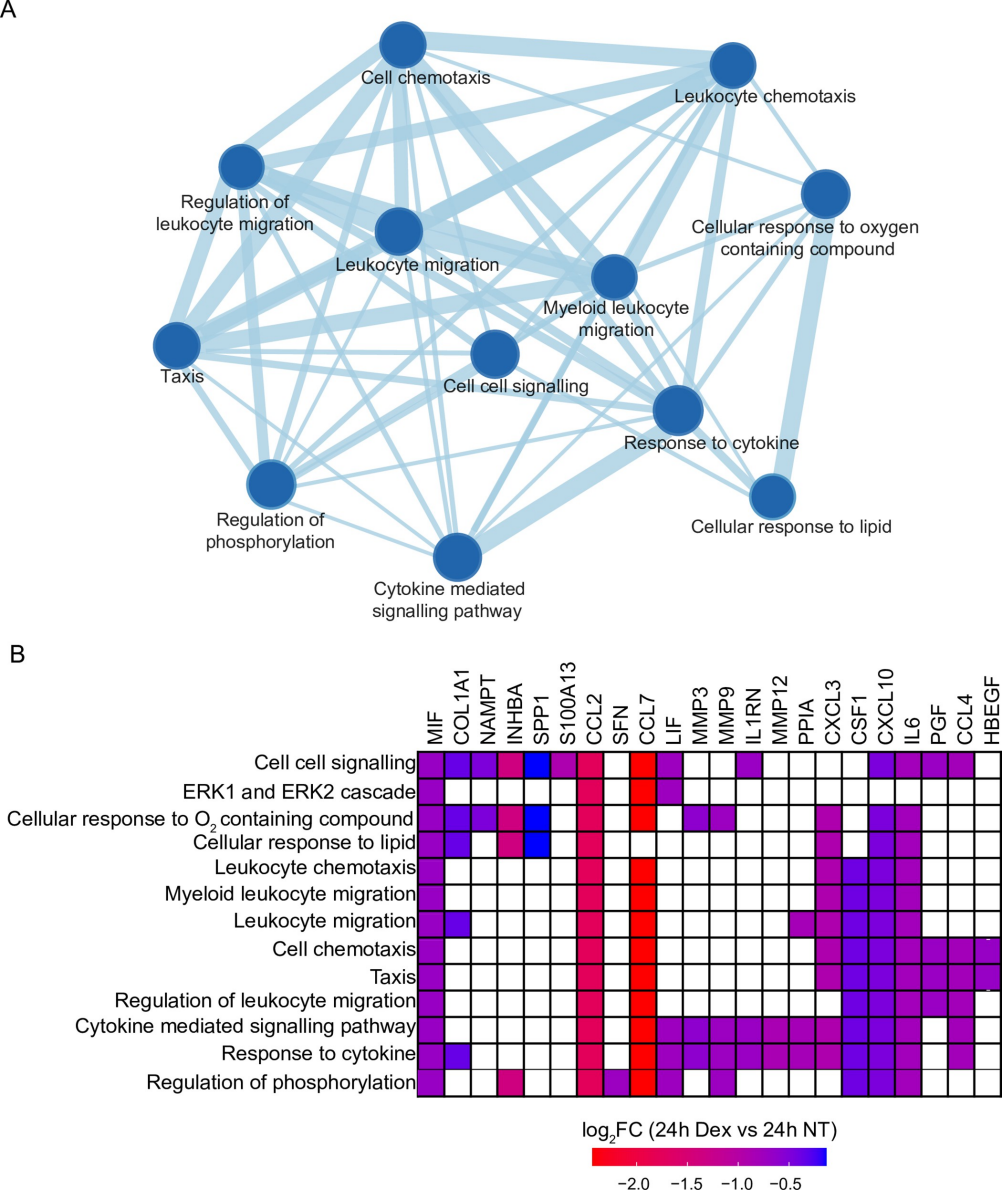
Gene set enrichment analysis (GSEA) of rat LSEC secreted proteins. GSEA leading-edge analysis including only proteins in the LSEC culture supernatants that were annotated as secreted proteins (n = 115) in the Human Protein Atlas [60]. (A) GSEA map showing processes that were significantly changed (all were downregulated) in the presence of Dex. The line thickness reflects the relative number of proteins that were affected. (B) Heatmap highlighting the leading-edge proteins contributing to the enrichment of the processes in A. The color of the heatmap corresponds to the amplitude of the log_**2**_ fold change between the Dex group and the non-treated (NT) control group.

Proteins in culture supernatants may stem from several sources, including Golgi-mediated secretory pathways, shedding of extracellular vesicles, histone release [59], fusion of lysosomes with the plasma membrane, or leakage from dead or dying cells. The LSEC secretome has not been fully characterized in any animal model, and the exact source of the proteins in the supernatant is not known. As primary LSEC cultures show substantial cell detachment/cell death [30, 31], we therefore focused the GSEA only on proteins annotated as secreted proteins according to the Human Protein Atlas [60]. This included 115 protein orthologs of the 1433 protein IDs in the LSEC supernatants. GSEA and leading-edge analysis of these secreted proteins showed that Dex led to downregulation of cell-cell signaling, and cellular responses to oxygen-containing compounds, chemotaxis, and leukocyte migration (Fig 5; S2 Fig).

**Fig 6:**
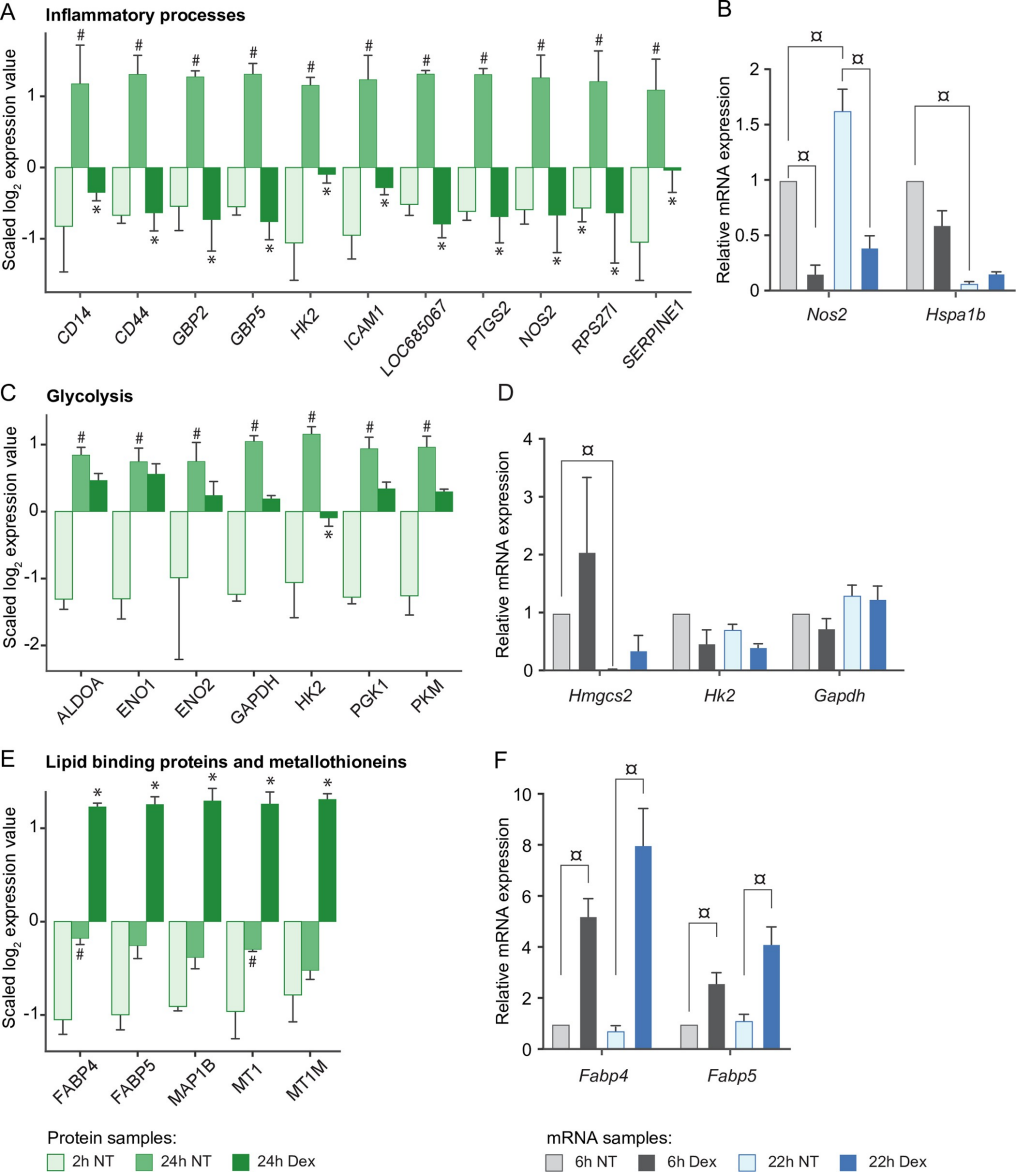
Differentially expressed proteins in selected processes affected by Dex in the cell-associated proteome of rat LSECs. Panels (A) and (C) show differentially expressed proteins contributing to the inflammatory-like phenotype (A), or glycolysis shift (C) in culture, while (E) shows the differential expression of lipid binding proteins (FABP4, FABP5), metallothioneins (MT1, MT1M), and microtubule-associated protein 1B (MAP1B). In panels (A), (C), and (E) the bars represent the Z-score value of the log_**2**_ normalized TMT reporter intensities for each protein per time point and treatment. Results are average values of 3 biological replicates. Error bars show SD. ^**#**^Significantly different between 24 h non-treated (NT) and 2 h NT cultures; *Significantly different between 24 h Dex-treated and 24 h NT cultures; FDR ≤ 0.05 and |log_**2**_ FC| ≥ 0.5. Error bars show SD. Panels (B), (D), and (F) show mRNA expression of genes linked to inflammatory responses (*Nos2*, *Hspa1b*) (B), glycolysis (*Hk2*, *Gapdh)* and ketogenesis (*Hmgcs2*) (D), and lipid binding (*Fabp4*, *Fabp5*) (F) in LSECs treated for 6 or 22 h with or without Dex (n = 5). Results are presented relative to the mRNA expression in 6h NT cultures for each gene. The combination of three genes, *Actb*, *Hprt* and *B2m*, was used as reference genes in all qPCR analyses. Error bars show standard error of the mean. ¤Significantly different between indicated groups, p < 0.05.

In the cell-associated proteome of LSECs, Dex significantly downregulated many proteins in the inflammatory response (Fig 6A), including intercellular adhesion molecule 1 (ICAM-1), CD14, CD44, nitric oxide synthase 2 (NOS2, or inducible nitric oxide synthase, iNOS), hexokinase 2 (HK2), and guanylate binding protein 2 and 5 (GBP2, GBP5). *Nos2* mRNA expression was further examined by qPCR after 6 and 22 h incubation of cells in the presence or absence of Dex. This showed an increased expression from 6 to 22 h in the non-treated cultures which was repressed by Dex at both time points (Fig 6B). We further measured the mRNA expression of heat shock family A member 1B (*Hspa1b*) by qPCR. The differential expression analysis of the proteome had shown upregulation of HSPA1B in all cultures at 24 h compared to 2 h, independent of Dex-treatment (Fig 3C). However, the mRNA expression of *Hspa1b* was significantly higher at 6 h than at 22 h, suggesting that this gene is activated early in culture.

Inflammation has been correlated to upregulation of glycolysis [61]. The functional enrichment analysis showed an upregulation of glycolysis in LSECs from 2 to 24 h both in non-treated and dex-treated cells (Fig 3A-module 2). However, several glycolytic proteins were less expressed in cells with Dex than with no Dex at 24 h (Fig 6C). This was significant only for HK2, and a similar trend was observed for *Hk2* expression at the mRNA level, although not significant (Fig 6D). The mRNA expression of *Hk2*, and glyceraldehyde-3-phosphate dehydrogenase (*Gapdh*) was examined by qPCR at 6 and 22 h (Fig 6D). The two enzymes did not show a significant change in mRNA expression from 6 to 22 h.

We further examined the LSEC mRNA expression of the rate-limiting enzyme in ketogenesis, 3-hydroxy-3-methylglutaryl-CoA synthase 2 (*Hmgcs2*). This enzyme was significantly downregulated from 6 to 22 h in culture, independent of Dex (Fig 6D), similar to the observed decline in HMGCS2 protein expression from 2 to 24 h (Fig 3B).

The proteins that showed the highest upregulation in the presence of Dex at 24 h were fatty acid-binding proteins (FABP4, FABP5), metallothioneins (MT1, MT1M), and the microtubule-associated protein 1B (MAP1B) (Fig 6E). The significant enhancement of *Fabp4* and *Fabp5* by Dex was validated by qPCR (Fig 6F).

### LSEC cytokine secretion and effect of Dex

**Fig 7:**
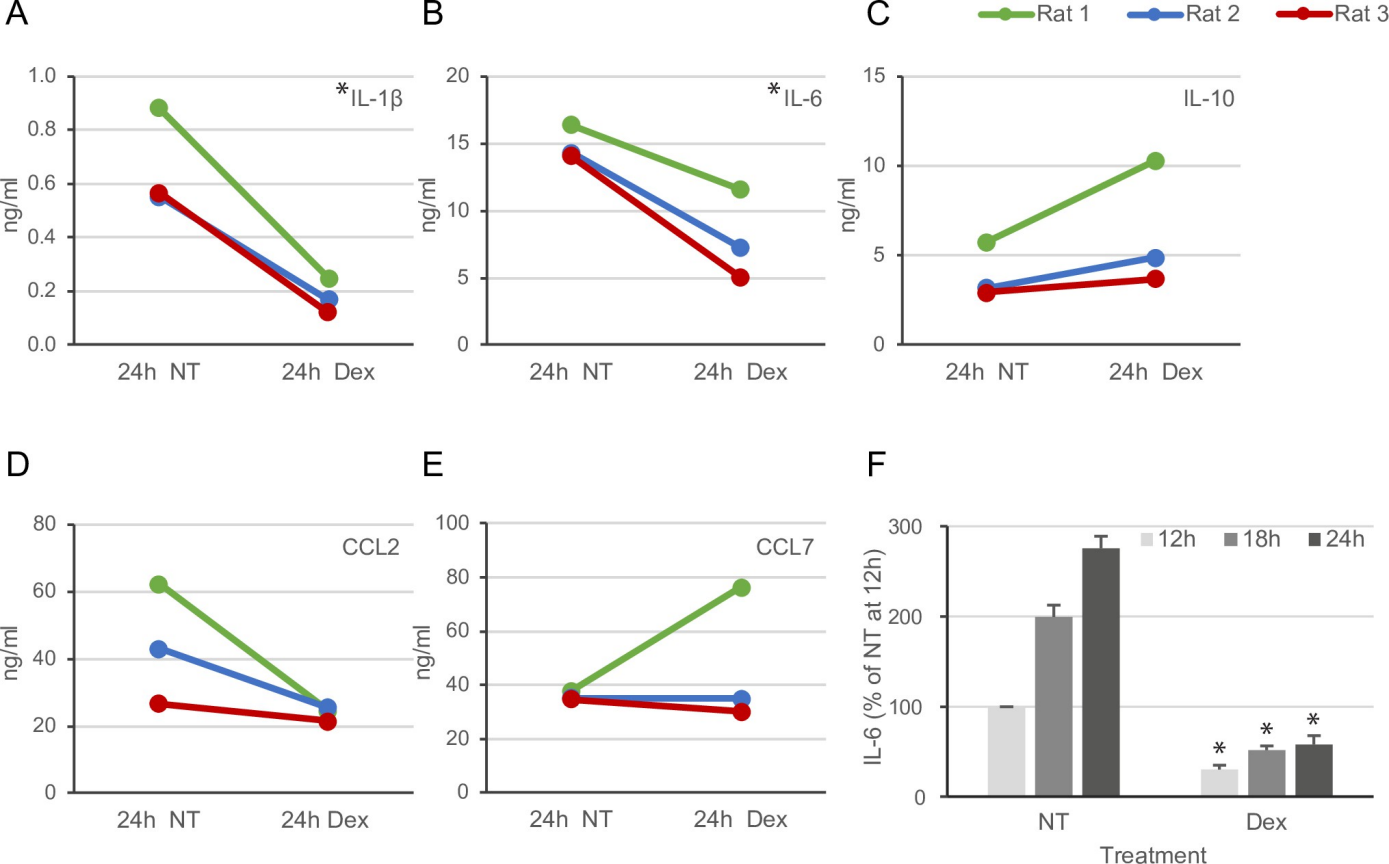
Cytokine production in rat LSECs treated with Dex. (A-E) Expression of selected cytokines and chemokines in supernatants of rat LSEC cultures ± 1 μg/ml Dex, measured by Luminex multiplex immunobead assay (n = 3). *Significantly different from 24 h non-treated (NT, Paired Samples T-test, p < 0.05). (F) IL-6 production (single ELISA) at 12, 18, and 24 h in rat LSEC cultures ± 1 μg/ml Dex (n = 3). *Significant difference between Dex and time-matched NT groups (one-way ANOVA, p < 0.05).

To validate some of the cytokines and chemokines that were affected by Dex in the quantitative TMT proteomics study (Figs 4, 5; S2 Fig), we used a multiplex immunobead assay to measure the production of IL-1β, IL-6, CCL2 (MCP-1), CCL7 (MCP-3), and IL-10 in supernatants harvested from LSEC cultures at 24 h (Fig 7A–7E). The LSEC secretion of IL-1β and IL-6 to supernatants was significantly downregulated by Dex, while the slightly reduced secretion of CCL2, and increased release of IL-10 was not significant. In contrast to the observation by proteomics, CCL7 was either similar or enhanced with Dex, compared to non-treated cells.

LSECs are important producers of IL-6 in the liver, and Dex is a well-known inhibitor of IL-6 [62]. To investigate more in detail LSEC production of IL-6 in early cultures and the effect of Dex, we incubated freshly isolated cells with Dex and measured IL-6 in supernatants at 12, 18, and 24 h (Fig 7F). The IL-6 production in the absence of Dex was 3 times higher at 24 h compared to 12 h post-seeding. Dex efficiently suppressed IL-6 production in LSECs at all time points.

### Changes in expression of LSEC signature receptors from 2 to 24 h

LSECs express a unique set of endocytosis receptors that are not expressed in most other endothelial cells and are regarded as LSEC signature receptors [8–10]. Of these, stabilin-1 (STAB1) and stabilin-2 (STAB2) [41, 63, 64], lymphatic vessel endothelial hyaluronan receptor 1 (LYVE1) [65], and C-type lectin domain family 4 member G (CLEC4G, LSECtin) [66] were significantly downregulated at 24 h both in the presence and absence of Dex (FDR ≤ 0.05, and |log_2_ FC| ≥ 0.5) (Fig 3B; S3A Fig). The expression of the mannose receptor (MRC1) [67, 68] was also reduced at 24 h (log_2_ FC = 0.44, FDR < 0.05; not significant, S1 Dataset, S3A Fig), and not affected by Dex, while two other LSEC signature receptors, FcγRIIb2 (FCGR2) [69], and C-type lectin domain family 4 member M (CLEC4M) [9, 70] showed a non-significantly reduced expression from 2 to 24 h in the absence of Dex (log_2_FC 0.45 and 0.23, respectively, FDR < 0.05, S1 Dataset) but preserved protein expression with Dex (S3A Fig). To gain more insight into the effect of time and Dex on LSEC endocytosis receptors we measured the mRNA expression of *Clec4g*, *Clec4m*, *Fcgr2b*, *Stab2* and *Lyve1* after 6 and 22 h by qPCR (S3B Fig). The results showed a significantly higher expression of *Fcgr2b* at 22 h in Dex-treated cells compared to 6 h (p < 0.05), suggesting that Dex has effect on the expression level of this receptor. The expression of the other receptors was not significantly different between groups.

### Dex improved cell viability in culture, and inhibited apoptosis but did not alter the declining LSEC endocytic activity

**Fig 8:**
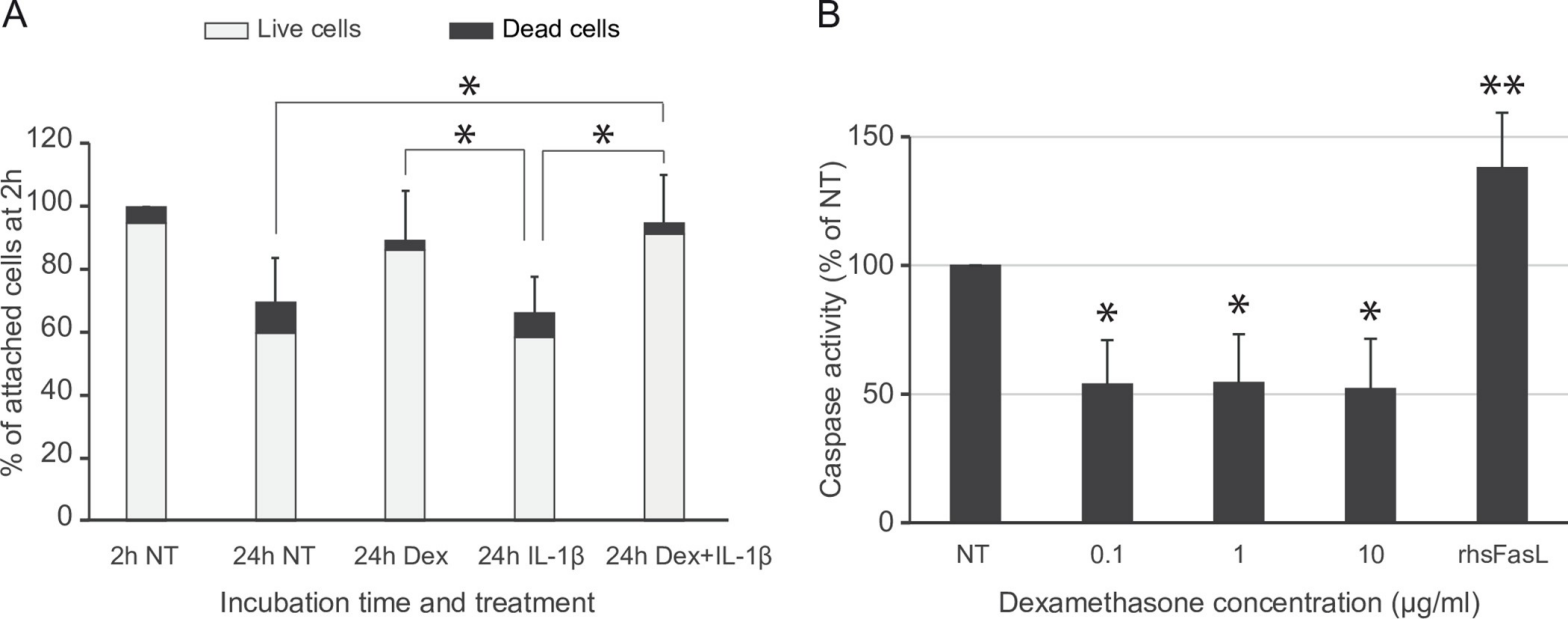
Effect of Dex on LSEC viability in culture. (A) Freshly isolated rat LSECs were seeded in similar densities and cultured for the indicated time points in 5% O_2_ and 5% CO_2_ in DMEM with serum-free supplements ± 1 μg/ml Dex, and/or 100 U/ml IL-1β. Cell viability was measured with a Live/Dead Cell Imaging Kit (Thermo Scientific) as described in Material and Methods. Results are presented as % of attached cells at 2 h (set as 100%) and are the average of 3 biological replicates. Error bars show SD. *Significant between groups (One-way ANOVA, Tukey HSD posthoc test, p < 0.05). (B) LSEC cultures were incubated with 0, 0.1, 1, or 10 μg/ml Dex for 24 h. Recombinant human Fas Ligand protein (rhsFasL, 100 ng/ml) was used as positive control and added to non-treated cultures 4h before the end of incubation time. Caspase 3/7 activity is shown in percent of the activity in non-treated LSECs without rhsFasL. Results are mean of 4 biological replicates; error bars show SD. *Significantly different from non-treated cultures and cultures with rhsFasL, **significantly different from non-treated cultures (one-way ANOVA, p < 0.05).

The effect of Dex on LSEC viability in culture was first tested in a Live/Dead assay. This showed a higher number of viable cells at 24 h in the presence of Dex (Fig 8A). The proteomics data (S1 Dataset) showed that caspase 3 was non-significantly suppressed in Dex-treated LSECs compared to non-treated cells at 24 h (log_2_FC = 0.27; FDR = 1.15E-06, S1 Dataset). We therefore performed a caspase 3/7 assay which detects active caspase-3 and caspase-7 in apoptotic cells. This showed that Dex at doses 0.1, 1.0, and 10 μg/ml significantly inhibited the caspase activity (Fig 8B), suggesting an anti-apoptotic effect of Dex on LSECs.

One of the core functions of LSECs *in vivo* is the highly effective endocytosis of many spent plasma proteins and other blood-borne macromolecules after binding of ligands to high affinity scavenger receptors and C-type lectins on the cells [8, 12]. The proteomics experiments showed a decline in culture of two of the major scavenger receptors in LSECs, namely stabilin-1 and stabilin-2 (S3 Fig). To test how LSEC endocytosis was affected by culture, and if Dex would influence on this function we examined the cellular uptake and subsequent degradation of the scavenger receptor ligand FSA. FSA is shown to bind to stabilin-1 and stabilin-2 in LSECs [41, 71], and is rapidly endocytosed by the cells both *in vivo* and *in vitro* [71, 72]. High endocytosis of FSA is also used as a functional marker for LSECs [12, 40, 73].

**Fig 9:**
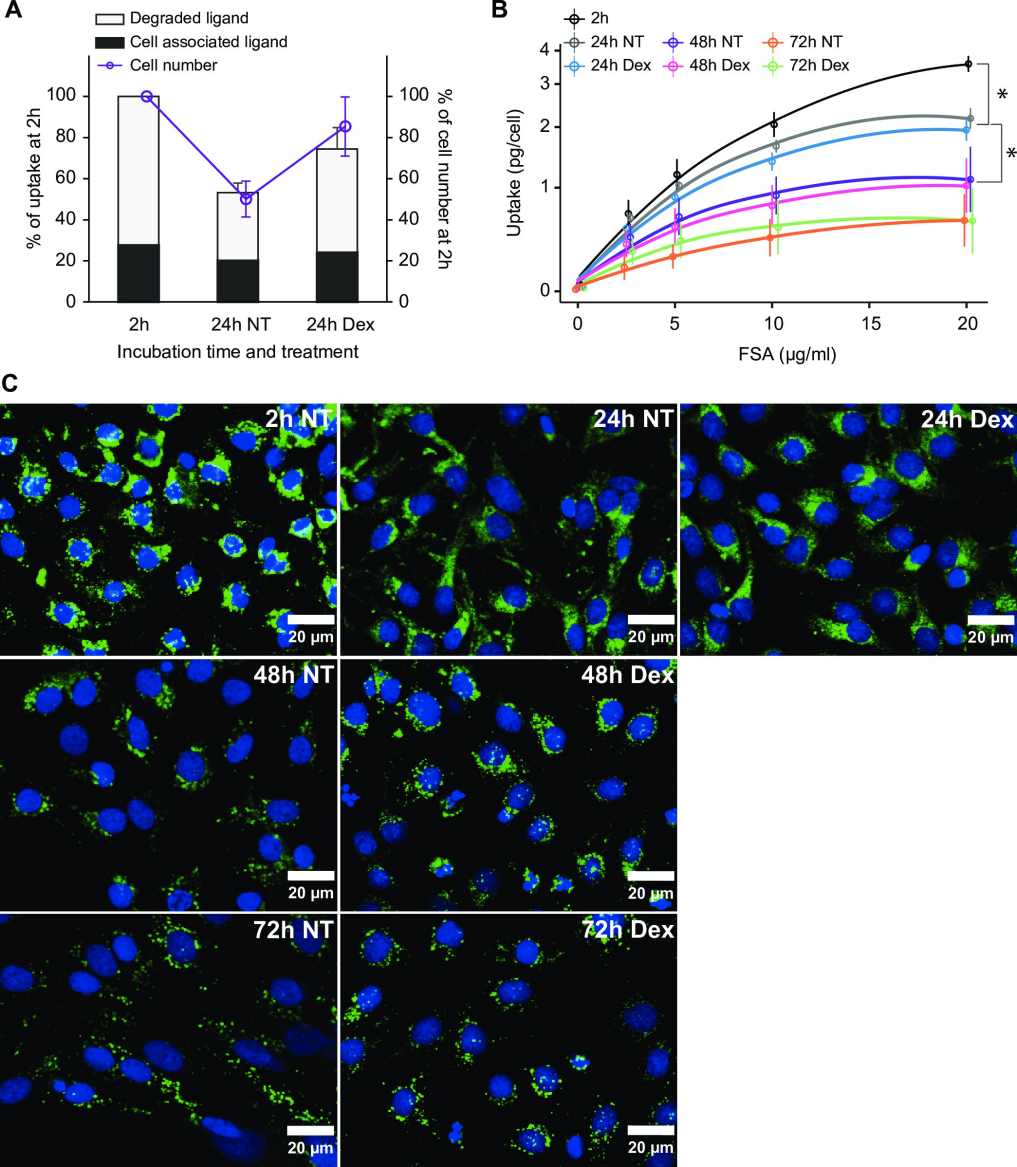
Effect of Dex on rat LSEC endocytosis in culture. (A) Endocytosis of trace amounts of ^**125**^I-labeled formaldehyde-treated serum albumin (^**125**^I-FSA) in primary LSEC cultures. The cells were cultured for 2 or 24 h ± Dex; then incubated with 0.1 μg/ml ^**125**^I-FSA for another 2 h. Ligand uptake per culture represents the sum of cell associated ligand (black part of bar), and degraded ligand in supernatant (grey part of bar) as described in Material and Methods. The total ligand uptake/culture was 67% (SD = 2.6%, n = 3) of added ligand in 2h cultures, and this is set as 100% in the figure, and uptake in the 24 h cultures given in % of this value. The number of adherent cells at 24 h post seeding is shown in % of the cell number at 2 h. The differences in uptake of ^**125**^I-FSA between groups were coherent with the number of attached cells in the cultures. NT, no treatment. (B) The LSEC capacity for endocytosis of FSA at different time in culture was measured by incubating the cells with 0.1 μg/ml ^**125**^I-FSA together with 0–20 μg/ml of non-labeled FSA for 2 h, starting the experiments at 2, 24, 48, or 72 h post seeding. Results were calculated as uptake in pg per cell (±SD, n = 3). *Significant for effect of time in culture (Area under the curve ANOVA, p < 0.01). The differences in endocytic capacity between Dex-treated cells and time-matched controls were not significant. (C) Uptake of fluorescent labeled FSA in rat LSEC culture at different time points post seeding. LSECs were cultured for up to 72 h in the presence or absence of Dex; then incubated with 20 μg/ml FITC-FSA (green fluorescence) for 30 min, washed, and incubated in ligand-free medium for 90 min, and fixed in 4% buffered formaldehyde. Nuclei were stained with DAPI (blue). The dose of Dex was 1 μg/ml in all experiments.

First, we measured the efficiency of uptake of trace amounts of radiolabeled FSA in LSECs. This was done by incubating LSEC cultures with ^125^I-FSA (approximately 0.1 μg/ml) for 2 h, starting at 2 or 24 h post-seeding, and measuring the proportion of the added ligand that had been internalized and processed by the cells during the 2 h incubation period (Fig 9A). The total uptake of ^125^I-FSA was 67% (± 2.6%, n = 3) of added ligand in the freshly plated (2 h) LSEC cultures. In 24 h old LSEC cultures, the uptake of ^125^I-FSA was reduced to 74% of this value in Dex-treated cells, and to 53% in non-treated cells (Fig 9A). The decrease in endocytosis of ^125^I-FSA with time in culture corresponded with the decrease in cell numbers in culture, suggesting that the higher uptake in the Dex-treated LSEC cultures may be explained by higher cell survival (Fig 8). The proportion of degraded ligand vs cell-associated ligand was almost identical at 2 and 24 h, with or without Dex, suggesting efficient degradation of internalized ligand in all groups.

Next, we measured the capacity of uptake of FSA in LSEC cultures, starting at different time points post seeding. The endocytic capacity, i.e. the maximum amount of ligand that can take up per LSEC in a given period of time, was measured by incubating LSEC cultures with ^125^I-FSA (0.1 μg/ml) together with increasing amounts (0–20 μg/ml) of non-labelled FSA for 2 h. The total amount of FSA taken up in each culture was then normalized to the number of cells in culture (Fig 9B). The endocytic capacity of LSECs significantly decreased with time in culture. There was no significant difference between the capacity of endocytosis of Dex-treated and non-treated LSECs in cultures at the same time points after plating.

Experiments with radiolabeled ligands only measure uptake per culture, from which average values for uptake per cell can be calculated. To examine if all cells in the cultures, or only a fraction of the cells were able to endocytose the ligand, we incubated LSECs with FITC-labelled FSA and examined uptake of ligand in the cells by fluorescence microscopy (Fig 9C). In these experiments the cells were first incubated with 20 μg/ml FITC-FSA for 30 min, followed by incubation of the cells for 1.5 h in medium without ligand. This procedure revealed that in all experimental groups nearly all viable cells in the LSEC cultures were able to internalize the ligand both at 2, 24, 48, and 72 h post-seeding, albeit the intensity of fluorescence in cells was lower in the long-term cultures. Dex treatment did not show visible impact on LSEC uptake of FITC-FSA compared to the non-treated control cultures at any given time point.

### Dex effects on the LSEC morphology in culture

**Fig 10:**
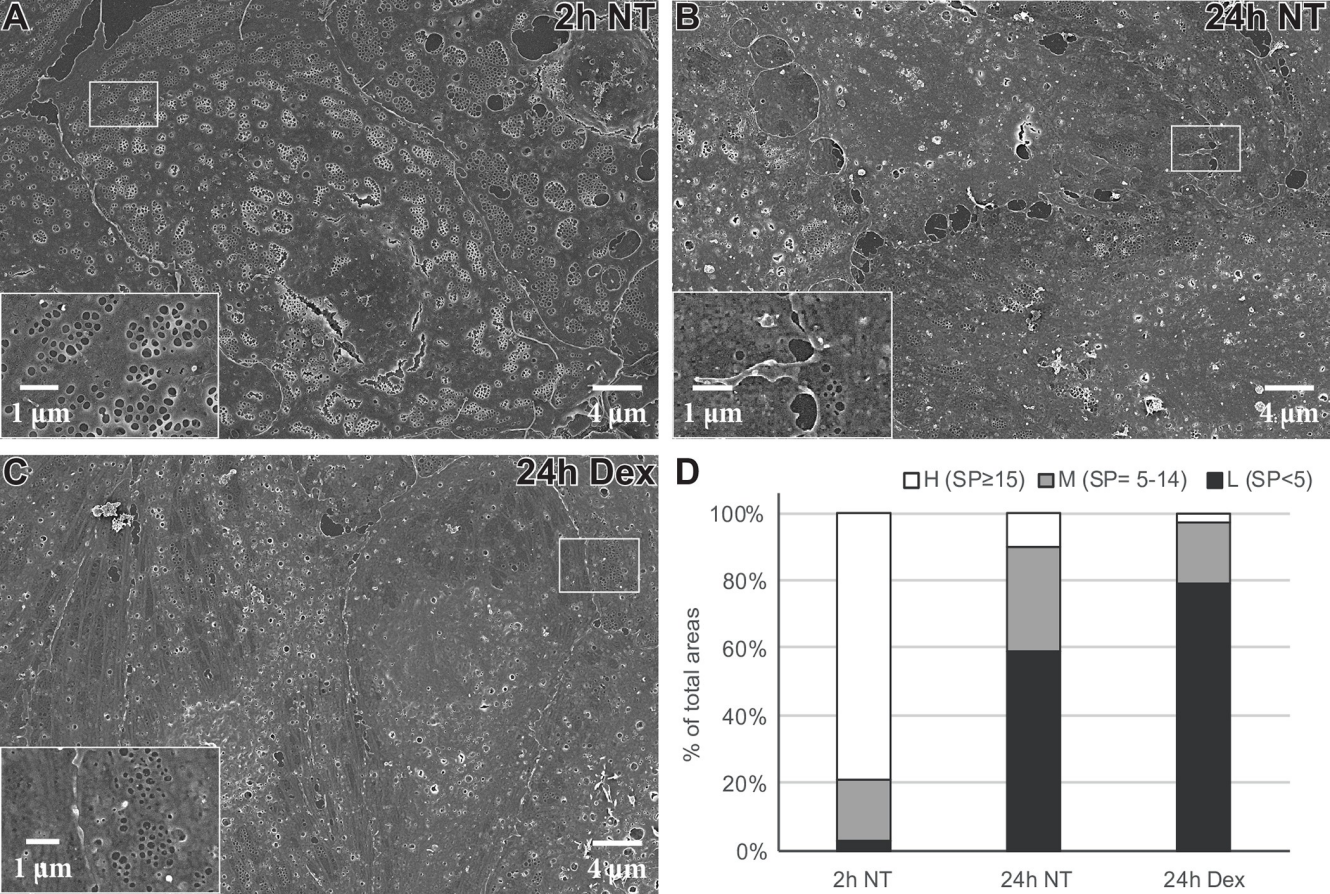
Effect of Dex on rat LSEC morphology in early primary cultures. (A-C) Scanning electron micrographs of LSECs cultured for the indicated time points in the presence or absence of 1 μg/ml Dex. Inserts show sieve plates (A-C) and cell borders (B, C) at higher magnification. (D) Extent of LSEC surface area with high density (H, ≥ 15 sieve plates (SP)/area), medium density (M, 5–14 SP/area), and low density (L, 0–4 SP/area) of fenestrae organized in SP in percent of total LSEC surface area, at the indicated time points and treatments. The number of SP was counted per 400 μm^**2**^ squares on images from randomly picked larger areas in the LSEC cultures. At least 100 squares were scored for each treatment in each experiment. The average values of 3 biological replicates are presented in the figure. The observed changes in % highly fenestrated areas were significant between 2 and 24 h (one-way ANOVA, p < 0.05, n = 3) but not between Dex- and non-treated groups at 24 h.

The effect of Dex on rat LSEC morphology and fenestration was analyzed by scanning EM (Fig 10). At 2 h post-seeding, LSECs were highly fenestrated with most fenestrae organized in sieve plates (Fig 10A). At 24 h, the number of sieve plates was markedly reduced, and in the cultures without Dex, the cells also showed an increased number of larger holes, *i*.*e*., gaps, which were located more at the edges of the cells, and between cells (Fig 10B). Dex-treated LSECs also showed marked defenestration at 24 h with loss of sieve plates. However, the cells had fewer gaps, less membrane ruffles or spikes, and the cell borders had a smooth appearance, with close contact between cells (Fig 10C), reflecting a less stressed phenotype. To estimate the reduction in sieve plates in cells at 24 h (± Dex) compared to 2 h, we counted the number of sieve plates per cell area (400 μm^2^) as described in Methods. At 2 h post seeding, approximately 79% of the cell surface had a high density of sieve plates (defined as ≥15 sieve plates/400 μm^2^). At 24 h, the percentage of cell surface area with a high density of sieve plates significantly dropped to 10% in LSECs cultured without Dex, and 3% in Dex-treated LSECs (Fig 10D). The difference in highly fenestrated areas between groups at 24 h was not significant.

## Discussion

In this study, we report how the rat LSEC proteome changes in early primary 2D culture, including analyses of both the cell-associated proteome and proteins in supernatants and the modulating effect of Dex. We used the TMT multiplexing strategy to accommodate all treatments belonging to a biological replicate in the same run to improve quantitation and consequently enhance the proteomics comparison applied. Having all samples from one experiment in one block simplifies the subsequent normalization between the runs and makes the quantitative comparisons between samples more valid [74], securing proteomics data that are suitable and sensitive to uncover culture-induced phenotypic and functional changes with accuracy and precision.

Deregulation of the LSEC morphology and scavenger functions in primary culture is well acknowledged in the literature [29–31, 40, 75]. However, the information about culture-induced changes at the level of processes and pathways is limited. Also, we have limited knowledge about the changes in the LSEC proteome in response to pro- and anti-inflammatory stimuli *in vitro*. We found that primary rat LSECs developed an activated phenotype in early culture with elevated expression of pro-inflammatory cytokines, chemokines, NOS2, and cell adhesion molecules, and enrichment in processes associated with cellular responses to cytokines and interferon-γ, and cell-cell adhesion. Upregulation of NOS2 is one of the hallmarks of endothelial inflammation which contributes to vascular dysfunction *in vivo* [76]. This was observed both in the presence and absence of IL-1β, and could be significantly downregulated by Dex.

LSECs have been previously shown to respond to inflammatory mediators such as lipopolysaccharide, tumor necrosis factor (TNF)-α, Toll-like receptor agonists, and other stimuli with enhanced IL-1β production [30, 39, 77–80], and to IL-1β inhibitors [39, 81]. However, rather few studies have been reported on direct IL-1β stimulation of cultured LSECs. These have focused mainly on effects on NO and H_2_O_2_ production, endocytosis, and cancer metastasis [38, 39, 82–84]. Two studies reported upregulation of LSEC endocytosis after short time (4–6 h) treatment with recombinant human IL-1β in rat and mouse LSECs [39, 82], while treatment for 6–24 h increased the NO production and decreased the endocytic activity in rat LSECs [38], and increased H_2_O_2_ production in mouse LSECs [83]. Another study in rat [84] reported that IL-1β alone could not induce iNOS (NOS2) in LSECs while IL-1β synergized with the interferon-γ -mediated upregulation of this enzyme, suggesting a complex regulation of NO production in LSECs. Notably, the different studies are not fully comparable—for instance there are differences in cell isolation procedures, use of medium, serum supplementation, and time point after culture establishment for start of stimulation. In the present study the treatment started at 2h after plating, which is earlier than in the other published studies on IL-1β effects on rat LSECs. Serum was omitted to be able to measure secreted proteins by TMT-quantitative proteomics. Serum is a complex compound with known and unknown effects on cells, as exemplified by the finding that it is a rich source of ligands for the LSEC scavenger receptors [85]. Hence, employing a defined medium was preferred.

The low effect of IL-1β on the rat LSEC proteome, which at first sight may seem surprising, can best be explained by the finding that the cells in early primary culture show a significant upregulation of inflammatory and immune regulatory processes/pathways, including production of proinflammatory cytokines such as IL-1β, and IL-6 [30]. These observations, together with the fact that LSECs are well-known producers of IL-1β and express receptor for IL-1β [81], which allow for autocrine signaling, suggest that the additional treatment with IL-1β produces little additional effects on the LSEC proteome *in vitro*.

The upregulation of inflammatory pathways and processes in early culture may be a result of what is referred to as “culture shock”, a phenomenon described in primary cells immediately after isolation, characterized by an increased production of reactive oxygen species and by the impairment of the antioxidant defense of primary cells [86]. The exposure to non-physiological factors and stimuli during tissue dissociation, cell purification, and culture establishment on a rigid surface [87] as well as lack of signals from other liver cells are also likely to contribute to an activated phenotype.

Glucocorticoids are potent anti-inflammatory drugs, and used in treatment of vascular diseases [36]. Dex substantially repressed the culture-induced stimulation of inflammatory and immune regulatory pathways in LSECs, as well as the expression of ICAM-1 and inflammatory mediators such as NF-κB1, NOS2, and other components of interferon responses. The analyses of the supernatant proteome showed that Dex suppressed rat LSEC production and release of pro-inflammatory cytokines (validated for IL-1β and IL-6) while increasing the secretion of the anti-inflammatory cytokine IL-10. However, the Dex effect on IL-10 secretion, as measured in the multiplex immunobead experiments was not significant. A previous study in mouse showed that pretreatment with Dex for 24 h significantly enhanced TLR-induced IL-10 mRNA expression in primary LSECs, while suppressing TLR-induced expression of TNFα and IL-6 [80]. Notably, the Dex-mediated modulation of the TLR-responses by glucocorticoids was different in hepatocytes, Kupffer cells and LSECs [80].

A central finding in our study was that Dex had a positive effect on LSEC survival *in vitro* and showed a significant inhibitory effect on apoptosis in functional assays. The anti-apoptotic effect was equal in all doses tested (0.1, 1, 10 μg/ml). Although glucocorticoid signaling is known to alter both pro-and anti-apoptotic genes that can either lead to apoptosis or cell survival depending on the cell type (reviewed in [88]), the proteome coverage in the present study was not deep enough to reveal the detailed regulation. Dex was shown to protect human umbilical vein endothelial cells (HUVEC) against apoptosis induced by serum-deprivation [89].

Cells in medium with Dex showed a different morphology than non-treated cells, with less membrane ruffles, smoother cell borders, and fewer gaps between cells. However, Dex was not able to prevent the culture-induced defenestration of LSECs, and the functional analysis of the cell-associated proteome did not reveal a significant effect on cytoskeleton organization. Rapid loss of LSEC porosity *in vitro* is a well-known phenomenon and is suggested to occur due to a lack of essential blood borne, or paracrine factors, such as VEGF [75, 90], interaction with other liver cells, changes in bioenergetic status, and the pronounced effect that a rigid substrate may have on the cells [30, 31, 75, 91]. Liver inflammation and fibrosis are commonly associated with LSEC defenestration [20, 21]. Interestingly, a study in mice on high-fat diet showed preserved fenestrations and largely preserved bioenergetics both in early and late stages of non-alcoholic fatty liver disease despite a proinflammatory LSEC phenotype [92]. This suggests a complex regulation of and influence by the microenvironment on LSEC fenestration both *in vitro* and *in vivo* (recently reviewed in [91]).

The functional enrichment analysis of our proteomics data revealed a significant shift in LSEC metabolism from 2 to 24 h in culture, with enhanced glycolysis and a reduction in pyruvate metabolism, citrate cycle, amino acid metabolism, oxidation-reduction processes, peroxisome, and PPAR-signaling. The proteome analysis further showed that several glycolytic enzymes were lower expressed (only significant for HK2) with Dex than without Dex at 24 h, suggesting that Dex to some extent counteracted the culture-induced upregulation of glycolysis. This change was not significant at mRNA level and specifically designed experiments on LSEC metabolism are needed to draw firm conclusions on the specific effect of Dex on the cell metabolism. Interestingly, while the proteomics analyses showed a significant upregulation of glycolysis from 2 to 24 h, the mRNA expression of *Hk2* and *Gapdh* did not significantly change from 6 to 22 h. This may suggest that the switch towards increased glycolysis in LSECs starts very early in culture.

The rate limiting enzyme of ketogenesis, *Hmgcs2*, was significantly downregulated with time in culture both in the proteomics experiments (Fig 3B) and at mRNA level (Fig 6D). This enzyme is known to be downregulated in high carbohydrate diet, and upregulated in carbohydrate fasting, and is regulated through PPAR-α [93]. The downregulation of *Hmgcs2* is in line with the observed metabolic switch in the cultured LSECs based on the proteomics data. It may be speculated that the use of standard DMEM high glucose medium along with insulin supplementation, which promotes glucose and amino acid uptake in the cells has impacted on the expression level of *Hmgcs2*.

There are few studies on LSEC metabolism [12]. LSECs are reported to generate most of their ATP and biosynthetic precursors from glutamine and fatty acid oxidation and less from glucose metabolism [94]. The glucose use increased five-fold in LSECs isolated from lipopolysaccharide-treated rats compared to saline-injected controls, primarily through increased glycolysis in the cells [95], which is in line with studies in other endothelial cells on the effect of inflammation on metabolism. Recently, evidence was presented that stimulation of glycolysis promotes inflammation in endothelial cells, while stimulation of oxidative phosphorylation and the pentose pathway have the opposite effect [96]. Our study, which was designed as a proteomics study cannot answer whether the metabolic shift towards enhanced glycolysis is due to the activated phenotype, or if the altered metabolism has triggered cell activation, as was reported in macrophages [97, 98]. However, the proteomics data suggests that activation of cells and changes in metabolic pathways occurs immediately or after few hours in culture, and that Dex represses the activation of cells while the effect of this drug on the LSEC metabolism needs to be examined with functional assays.

The proteins that showed the highest upregulation in the presence of Dex were the fatty acid binding proteins FABP4 and FABP5. The finding was validated by qPCR which showed significantly higher mRNA expression of *Fabp4* and *Fabp5* after 6 and 22 h treatment with Dex, as compared to time-matched controls. Fatty acid binding proteins are small lipid chaperones that are ubiquitously expressed, with different isoforms expressed in different tissues, with some overlap [99, 100]. Co-expression of FABP4 and FABP5 is reported in subsets of endothelial cells and macrophages [100, 101], and both proteins were recently reported in primary rat and mouse LSECs [102, 103]. Another study in rat which compared the transcriptomes and non-labelled proteomes of freshly plated LSECs (SE-1-MACS purified) with Kupffer cells (CD11b/c MACS purified), showed a significantly higher expression of FABP4 and FABP5 in LSECs compared to Kupffer cells ([9], data included in Supplemental file 2 of the paper).

Interestingly, *FABP4* and *FABP5* are two out of four genes, including also *VWF/a1*, shown to distinguish damaged LSECs from healthy LSECs at transcriptome level in acute liver injury induced by CCl_4_ in mouse, and in chronic liver disease of various etiologies in human and mouse [102]. The four gene signature of damaged LSECs was consistently observed in single cell RNA sequencing and microarray datasets from normal liver and from patients with alcoholic and non-alcoholic liver disease, hepatocellular carcinoma, and liver fibrosis due to hepatitis B. Another recent study showed that FABP4 induced in LSECs in mice after bile duct ligation can promote LSEC capillarization by activation of Hedgehog signaling and stimulate hepatic stellate cells to increase the production of transforming growth factor β1 [103]. The present and previous studies have shown that primary LSECs in culture rapidly change towards a more dysfunctional phenotype [29–31]. Thus, the increased FABP4/5 expression observed in our study supports the reported observation [102] that they may be markers of damaged LSECs. It was therefore a surprise that Dex exposure significantly increased the LSEC protein expression of FABP4 and FABP5 and at the same time increased LSEC survival, and reduced LSEC apoptosis in culture.

Upregulation of FABP4 by Dex has been reported in the Raw mouse macrophage cell line and in peritoneal macrophages from wild-type mice fed chow with Dex alone or Dex together with statins for 2 weeks [104]. Interestingly, statins synergized with the Dex-induced FABP4 expression in the mouse macrophages while statins alone reduced FABP4 expression. Increased macrophage FABP4 expression is linked to atherosclerosis, and co-treatment of statins and Dex, but not Dex alone, exacerbated high-fat diet-induced atherosclerosis in apoE-deficient mice [105]. FABP4 and FABP5 are regulated through PPAR receptors and demonstrate synergistic effects in regulation of metabolic and inflammatory responses in adipocytes and macrophages [101]. FABP4 is further reported to have an anti-apoptotic function in endothelial cells (HUVEC) [106], while FABP5 functioned as a positive regulator of PPARδ in HUVEC cultured with serum while inhibiting the activity of PPARδ in serum deprivation and reduced cell death by apoptosis [101]. Notably we have used a serum-free medium in our study, and more studies are needed to elucidate the function and regulation of FABP4 and FABP5 in LSECs, as well as the influence on LSEC viability *in vitro* and *in vivo*.

The metallothionein proteins MT1 and MT1M were also highly upregulated by Dex in rat LSECs in the present study (Fig 6E). Metallothionein is the part of thiol antioxidants that is involved in heavy metal detoxification and is elevated in response to oxidative stress, controlled by the Nrf2-antioxidant response element signaling pathway [107, 108]. The MT genes have a glucocorticoid responsive element upstream which explains the upregulation by Dex, while the major downstream effect of MT expression result in modulation of transcription of NF-kB. MT scavenging of free radicals may directly influence cell survival, growth, and differentiation of cells [107]. Upregulation of MT1 and MT1M may therefore represent part of the mechanism behind the positive effect of Dex on LSEC viability in our experiments.

In accordance with [29], membrane receptors that are constitutively expressed in LSECs and regarded as LSEC signature genes/proteins were significantly downregulated in the rat LSEC cultures from 2 to 24 h. These included LYVE-1 (LYVE1), stabilin-1 (STAB1), stabilin-2 (STAB2), and CLEC4G. Although Dex improved cell viability and suppressed cell activation, Dex did not affect the expression of these receptors. LYVE-1 is a hyaluronan binding protein constitutively expressed by lymphatic vascular endothelial cells, LSECs, and spleen sinusoidal endothelial cells [65, 109], and is often downregulated during liver inflammation and in fibrosis in both humans and animal models [110]. However, in acute liver injury caused by CCl_4_ in mice, LYVE-1 was found to be upregulated both at mRNA and protein level (immune histochemistry of liver sections) [102].

The two stabilins are homologous protein [63]. Both are broad-spectrum scavenger receptors with partly overlapping ligand binding properties [10, 12, 63]. Stabilin-2 has been suggested as the major work-horse in LSEC clearance of spent plasma proteins, and soluble waste material from connective tissue turnover, possibly sharing part of this task with stabilin-1 [7, 8]. In the present study we observed a significant, time-dependent downregulation of endocytosis of the scavenger receptor ligand FSA in rat LSECs, which was not changed by Dex. Stabilin-2 is the major endocytosis receptor for FSA in LSECs, and a stabilin-2 blocking antibody inhibited more than 60% of endocytosis of radiolabeled FSA in rat LSECs [41]. FSA can also be taken up by stabilin-1 [71] but the relative contribution of this receptor is not known. Downregulation of LSEC Stabilin-2 expression is observed in acute and chronic liver injury in mouse and human [10, 102], and in old rat liver [111], suggesting that the LSEC scavenger function may also be impaired in these conditions. In accordance with this, the LSEC capacity for endocytosis of FSA was significantly reduced in cells from old rats compared to young animals [112].

The C-type lectin CLEC4G (LSECtin) has a postulated role in viral infection of cells and interacts with envelope glycoproteins on Japanese encephalitis virus [113], lymphocytic choriomeningitis virus [114], ebolavirus [115], and SARS-CoV-1 spike glycoproteins [115]. As for the stabilins, we observed a significant time-dependent decline in CLEC4G in the LSEC cultures, and a non-significant downregulation of mRNA expression by qPCR. This protein is also downregulated in acute and chronic liver injury and fibrosis in mouse and human, and has been suggested as a potential marker for damaged LSECs [102].

Interestingly, the culture-induced (non-significant) downregulation of FCGR2 (FcγRIIB) in the LSEC proteome at 24h was not observed in the cultures with Dex (S3 Fig), and this observation was supported by the qPCR experiments, which showed a significant upregulation of *Fcgr2b* mRNA expression in Dex-treated cultures at 22 h vs 6 h (S3 Fig). LSECs express FcγRIIB2 [69], which is a splice variant of *Fcgr2b*, and the cells are the main carrier of this receptor in liver [116]. Notably, our proteomics data did not distinguish between splice variants. FcγRIIB2 is the only Fc receptor in LSECs and mediates uptake of small, soluble immune complexes in the cells [69]. Several reports show a decline in the expression of this receptor in primary LSEC culture [29, 31], as well as in liver fibrosis, while in the CCl_4_-model of acute liver injury in mice this receptor was downregulated only during the early phase (day 1–3) of acute injury, then restored at day 7 [102].

Among the receptor proteins that were upregulated in culture and significantly suppressed by Dex were CD14 that regulates LPS-induced endocytosis of TLR 4 and modulates responses in monocytes and LSECs [117], and CD44 which is a receptor for matrix molecules such as hyaluronan, osteopontin, and matrix metalloproteinases and is involved in lymphocyte activation and homing [14].

Our study lacks sufficient proteome coverage to look at culture- and Dex-induced effects on LSEC transcription factors. However, others have reported that GATA4 [29], together with c-MAF and MEIS2 [118], are critical regulators of the LSEC phenotype, and downregulation of these factors promoted the dedifferentiation of LSECs in culture [29, 118]. In [29] down-regulation of stabilin-1, stabilin-2, FcγRIIb2, and LYVE-1 in early rat LSEC cultures was observed in parallel with changes in growth and transcription factors important for the LSEC phenotype, and after 42 h the cells became more like lung microvascular endothelial cells.

In conclusion, our study extends the knowledge about culture-induced changes in rat LSEC metabolism, receptor expression, morphology, and function in culture. The supplementation of Dex to the culture system significantly improved LSEC survival and repressed the culture-induced upregulation of pro-inflammatory proteins and mediators but was unable to sustain the LSEC scavenger function and cell fenestration.

## Supporting information

S1 Dataset The processed proteome–whole dataset. The excel file shows the cellular proteins, and proteins in supernatants that were expressed in the samples from the rat LSEC cultures included in the analyses and visualization. The excel workbook consists of two worksheets named “Rat_LSEC_TMT6plex_Cell_Prot”, and “Rat_LSEC_TMT6plex_Supernat_Prot”. The former sheet contains the list of proteins from the cell lysates, the corresponding normalized TMT intensities, and the output of the differential expression analysis with edgeR, while the latter worksheet contains similar information for the proteins in the LSEC culture supernatants.
(XLSX)

S1 Fig IL-6 production in rat LSEC cultures stimulated with human recombinant interleukin-1β. For titration of dose of IL-1β for the proteomics experiments freshly prepared LSEC cultures were incubated with 0, 10, 100, 1000 U/ml of IL-1β for 6, 18 and 24 h, and the IL-6 concentration in the supernatants were measured by ELISA. Results are presented as fold change (FC ± standard error of the mean) compared to 6 h level. Number of biological replicates, n = 3.
(PDF)

S2 Fig Pro- and anti-inflammatory mediators in rat LSEC culture supernatants, and effects of dexamethasone. The figure shows the differential expression (Z-score value of the log_2_ normalized TMT intensities) of selected proteins in supernatants (n = 3). These are leading-edge proteins contributing to the enrichment of the processes shown in Fig 5. NT, non-treated; Dex, dexamethasone (1 μg/ml). *Significantly altered between the two groups (FDR ≤ 0.05 and |log_2_ FC| ≥ 0.5). Error bars show SD.
(PDF)

S3 Fig Expression of endocytosis receptors in rat LSEC culture. The figure in A shows the differential expression (Z-score value of the log_2_ normalized TMT intensities) (n = 3) of membrane receptors characteristic for the LSEC phenotype [9, 10], and the endothelial cell marker MCAM (CD146). NT, non-treated; Dex, dexamethasone. #Significantly altered at 24 h compared to 2 h NT (FDR ≤ 0.05 and |log_2_ FC| ≥ 0.5). Error bars show SD. Figure B shows mRNA expression of *Clec4g* (n = 5), *Clec4m* (n = 4), *Fcgr2b* (n = 5), *Stab2* (n = 4), and *Lyve1* (n = 5) in LSECs treated for 6 or 22 h with or without Dex (1 μg/ml). Results are presented relative to the mRNA expression in non-treated cultures at 6 h for each gene. The combination of *Actb*, *Hprt* and *B2m*, was used as reference genes in all qPCR analyses. *Significantly altered between indicated groups, p < 0.05. Error bars show standard error of the mean.
(PDF)

S1 Table Information on targets and primers used for qPCR experiments. (PDF)

S2 Table Performance of the qPCR assays. (PDF)
