# Supplementary figures and images for: Changes in the proteome and secretome of rat liver sinusoidal endothelial cells during early primary culture and effects of dexamethasone
Source: PLoS One. 2022 Sep 2;17(9):e0273843. doi: 10.1371/journal.pone.0273843 (PMC9439253; doi:10.1371/journal.pone.0273843)

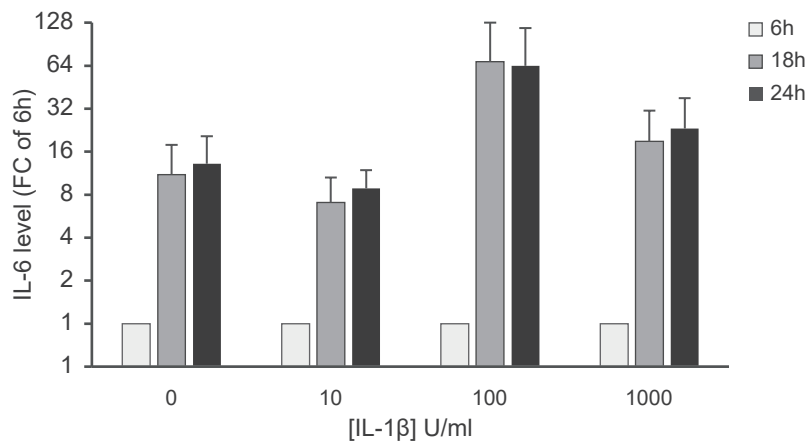

Supplement: S1 Fig — For titration of dose of IL-1β for the proteomics experiments freshly prepared LSEC cultures were incubated with 0, 10, 100, 1000 U/ml of IL-1β for 6, 18 and 24 h, and the IL-6 concentration in the supernatants were measured by ELISA. Results are presented as fold change (FC ± standard error of the mean) compared to 6 h level. Number of biological replicates, n = 3. (PDF) [file pone.0273843.s002.pdf]

A

Chemokines, cytokines and cytokine receptors

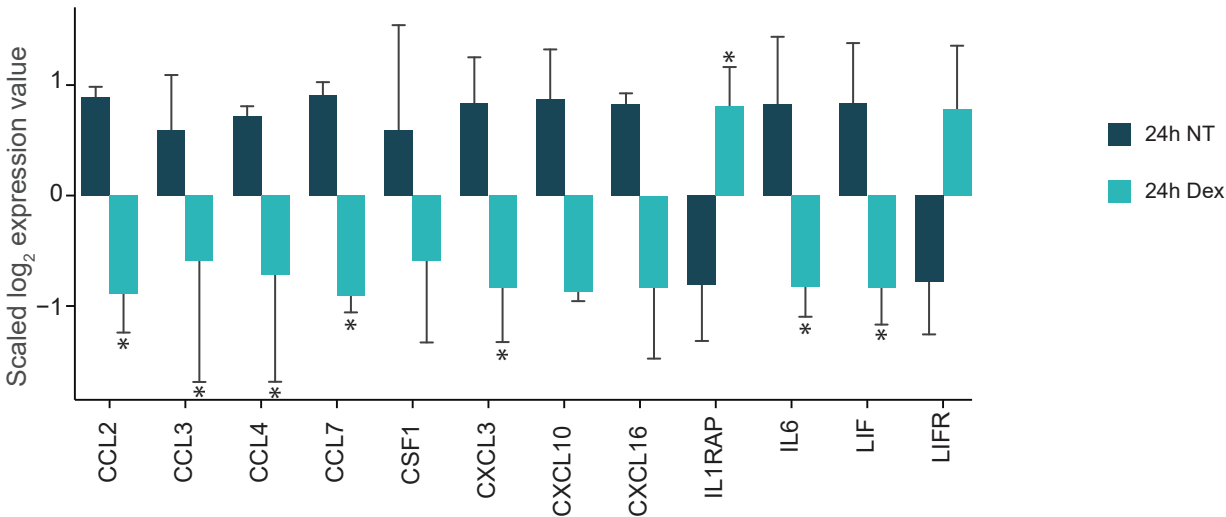

B

Matrix metalloproteases and inhibitors

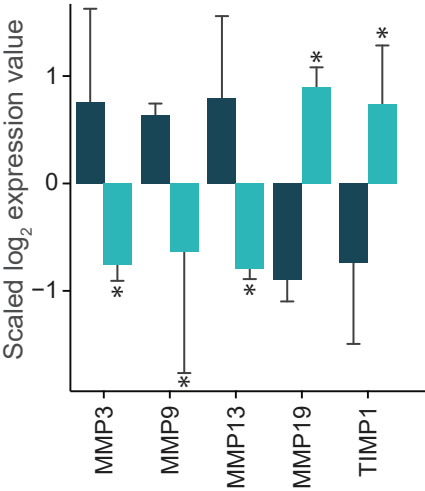

Supplement: S2 Fig — The figure shows the differential expression (Z-score value of the log2 normalized TMT intensities) of selected proteins in supernatants (n = 3). These are leading-edge proteins contributing to the enrichment of the processes shown in Fig 5. NT, non-treated; Dex, dexamethasone (1 μg/ml). *Significantly altered between the two groups (FDR ≤ 0.05 and |log2 FC| ≥ 0.5). Error bars show SD. (PDF) [file pone.0273843.s003.pdf]

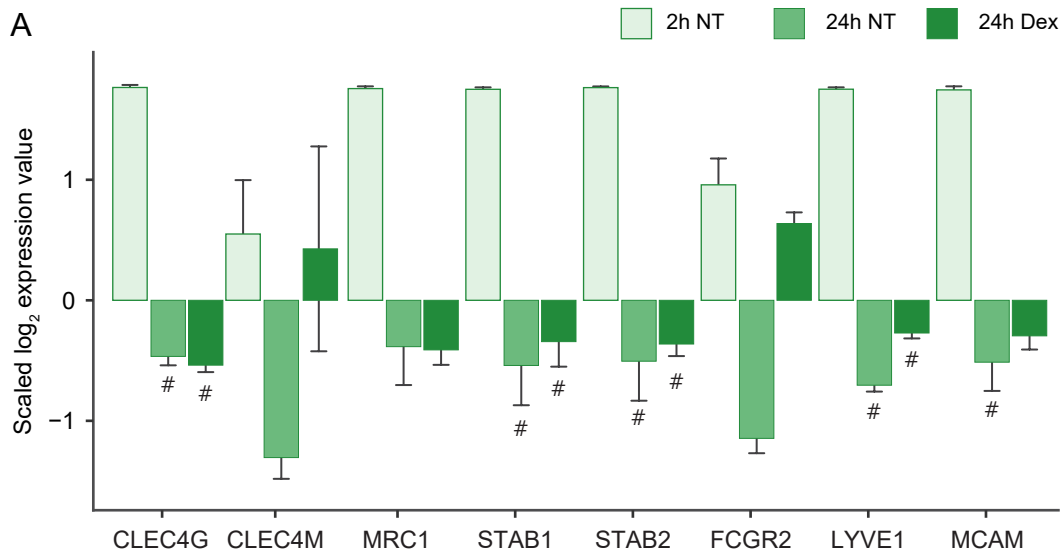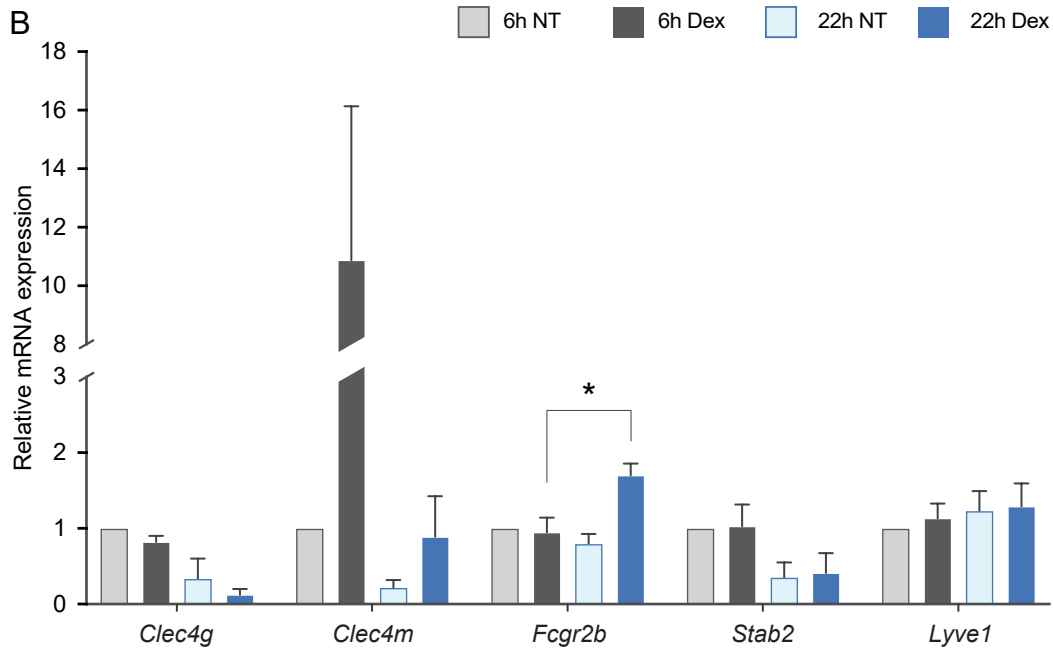

Supplement: S3 Fig — The figure in A shows the differential expression (Z-score value of the log2 normalized TMT intensities) (n = 3) of membrane receptors characteristic for the LSEC phenotype [9, 10], and the endothelial cell marker MCAM (CD146). NT, non-treated; Dex, dexamethasone. #Significantly altered at 24 h compared to 2 h NT (FDR ≤ 0.05 and |log2 FC| ≥ 0.5). Error bars show SD. Figure B shows mRNA expression of Clec4g (n = 5), Clec4m (n = 4), Fcgr2b (n = 5), Stab2 (n = 4), and Lyve1 (n = 5) in LSECs treated for 6 or 22 h with or without Dex (1 μg/ml). Results are presented relative to the mRNA expression in non-treated cultures at 6 h for each gene. The combination of Actb, Hprt and B2m, was used as reference genes in all qPCR analyses. *Significantly altered between indicated groups, p < 0.05. Error bars show standard error of the mean. (PDF) [file pone.0273843.s004.pdf]
