# Supplementary material for: Changes in the proteome and secretome of rat liver sinusoidal endothelial cells during early primary culture and effects of dexamethasone
Source: PLoS One. 2022 Sep 2;17(9):e0273843. doi: 10.1371/journal.pone.0273843 (PMC9439253; doi:10.1371/journal.pone.0273843)
Supplement: S1 Table — (PDF) [file pone.0273843.s005.pdf]

S1 Table. Information on targets and primers used for qPCR experiments

Symbol	Accession number	Primers	Sequence	Length	Amplicon	Range
Fabp4	NM_053365.2	Forward	CACCTGGAAGAGAA CTCCTTG	21	117	52-72
		Reverse	GAAGCCAACTCCCA CTTCTT	20		168-149
Fabp5	NM_145878.2	Forward	AGGACCTGGAAGG GAAGTGG	20	87	51-70
		Reverse	TCCTAAGAGCCAGC CCTACT	20		137-118
Hk2	NM_012735.2	Forward	AACCAAGTGCAGAA GGTTGA	20	115	245-264
		Reverse	TGGTAGCTCCTAGC CCTTT	19		359-341
Hmgcs2	NM_173094.2	Forward	CTAGCTCGGCTGAT GTTCAAT	21	100	965-985
		Reverse	CTT CCA GCT TTA GAC CCT TGA	21		1064-1044
Stab2	NM_001246357.2	Forward	CTCTAACCATCAAG ACGGAGTG	22	121	181-202
		Reverse	TCAAGCGAATACCT GCAATCT	21		301-281
Clec4g	XM_039089900.1	Forward	GAGTTCCCTGAAGG ATGATGTC	22	104	294-315
		Reverse	CCTTAGCCTGTGTG TCCTTAAA	22		397-376

Clec4m	NM_001170397.1	Forward	CCAGCTGACAGACGAACTTAT	21	115	437-457
		Reverse	CCTGGATAGGAGTTCAACCTTC	22		551-530
Fcgr2b	NM_175756.3	Forward	ATCCTGGTATCCTTGGTCTATCT	23	99	800-822
		Reverse	GGTGATTGTATTCTCCACCTCAT	23		898-876
Lyve1	NM_001106286.1	Forward	GTGCAAGACCTTTCCATTTCTC	22	111	438-459
		Reverse	CAGAACCTTACAGACCTCCTTG	22		548-527
Nos2	NM_012611.3	Forward	CTACCAAGGTGACCTGAAAGAG	22	82	229-250
		Reverse	TGTTGTTGGGCTGGGAATAG	20		310-291
Hspa1b	NM_001329896.1	Reverse	ACTTGTCCAGCACCTTCTTC	20	95	1897-1878
		Forward	CGAGTCCTACGCCTTCAATATG	22		1803-1824

Targets and primers for *Gapdh*, *Actb, Hprt,* and *B2m* were from TATAA Biocenter (Cat. No A103).
