# Supplementary material for: Changes in the proteome and secretome of rat liver sinusoidal endothelial cells during early primary culture and effects of dexamethasone
Source: PLoS One. 2022 Sep 2;17(9):e0273843. doi: 10.1371/journal.pone.0273843 (PMC9439253; doi:10.1371/journal.pone.0273843)
Supplement: S2 Table — (PDF) [file pone.0273843.s006.pdf]

S2 Table. Performance of the qPCR assays

Primer pair	[Primer] (nM)	Slope	Efficiency	R^2	Y-Intercept
Fabp4	600 Forward<br>600 Reverse	-3.5526	1.91	1	37.01
Fabp5	600 Forward<br>600 Reverse	-3.4366	1.95	1	35.83
Hk2	600 Forward<br>600 Reverse	-3.5256	1.92	1	35.89
Hmgcs2	600 Forward<br>600 Reverse	-3.403	1.97	1	34.51
Stab2	600 Forward<br>600 Reverse	-3.3992	1.97	1	35.71
Clec4g	600 Forward<br>600 Reverse	-3.4738	1.94	1	35.35
Clec4m	600 Forward<br>600 Reverse	-3.4583	1.95	1	34.9
Fcgr2b	600 Forward<br>600 Reverse	-3.5257	1.92	1	35.51
Lyve1	600 Forward<br>600 Reverse	-3.5269	1.92	0.99	36.01
Nos2	600 Forward<br>600 Reverse	-3.5833	1.90	0.99	35.039

Hspba1	600 Forward<br>600 Reverse	-3.4928	1.93	1	35.18

The qPCR assay performance was determined by performing calibration curves with gBlock Gene fragments (Integrated DNA Technologies) specifically designed for the target sequences. The linear range to perform calibration curves was from 10 copies per well to 10E6 copies per well. Slope, y-intercept, PCR efficiency and R^2 from the calibration curves are shown.
